# Reconstructing genetic histories and social organisation in Neolithic and Bronze Age Croatia

**DOI:** 10.1038/s41598-021-94932-9

**Published:** 2021-08-18

**Authors:** Suzanne Freilich, Harald Ringbauer, Dženi Los, Mario Novak, Dinko Tresić Pavičić, Stephan Schiffels, Ron Pinhasi

**Affiliations:** 1 Department of Evolutionary Anthropology, University of Vienna, 1090 Vienna, Austria; 2 Department of Archaeogenetics, Max Planck Institute for the Science of Human History, 07745 Jena, Germany; 3 Department of Genetics, Harvard Medical School, Boston, MA 02115 USA; 4 Department of Human Evolutionary Biology, Harvard University, Cambridge, MA 02138 USA; 5 Department of Human Genetics, University of Chicago, Chicago, IL 60637 USA; 6 Kaducej Ltd., 21000 Split, Croatia; 7 Centre for Applied Bioanthropology, Institute for Anthropological Research, 10000 Zagreb, Croatia

**Keywords:** Genomics, Biological anthropology, Archaeology

## Abstract

Ancient DNA studies have revealed how human migrations from the Neolithic to the Bronze Age transformed the social and genetic structure of European societies. Present-day Croatia lies at the heart of ancient migration routes through Europe, yet our knowledge about social and genetic processes here remains sparse. To shed light on these questions, we report new whole-genome data for 28 individuals dated to between ~ 4700 BCE–400 CE from two sites in present-day eastern Croatia. In the Middle Neolithic we evidence first cousin mating practices and strong genetic continuity from the Early Neolithic. In the Middle Bronze Age community that we studied, we find multiple closely related males suggesting a patrilocal social organisation. We also find in that community an unexpected genetic ancestry profile distinct from individuals found at contemporaneous sites in the region, due to the addition of hunter-gatherer-related ancestry. These findings support archaeological evidence for contacts with communities further north in the Carpathian Basin. Finally, an individual dated to Roman times exhibits an ancestry profile that is broadly present in the region today, adding an important data point to the substantial shift in ancestry that occurred in the region between the Bronze Age and today.

## Introduction

Croatia in southeast Europe is home to a diverse landscape of contiguous ecoregions, with steep mountains separating the eastern Adriatic coast from the temperate Pannonian Plain in the north. Its central location at the interface of Central Europe, the Balkan Peninsula and the Mediterranean has long promoted it as a conduit to Anatolia, the Aegean and the steppe region as far as the Black Sea, with the northern lowlands connecting it to passes through the Carpathian Basin to Europe beyond^1^. Thus, this region was a significant corridor for the first migrating farmers from western Anatolia, who dispersed throughout the rest of Europe via inland and littoral routes along the Danube River and eastern Adriatic coast respectively^2,3^. While this region is important for understanding population and cultural transitions in Europe, limited availability of human remains means that in-depth knowledge about the genetic ancestry and social complexity of prehistoric populations here remains sparse.

Previous studies have demonstrated genetic discontinuity following the Mesolithic in west Eurasia, associated with the migration of early farmers and the spread of agriculture^2,3^. Published genome-wide data from a small number of ancient individuals from present-day Croatia have shown how Neolithic and Copper Age genomes share similar ancestry with early farmers from Anatolia, but some Copper Age and coastal Bronze Age individuals display additional ancestry associated with steppe pastoralist populations^2^ that dispersed into Europe during the third millennium BCE. The beginnings of social complexity in southeast Europe has also been an area of intensive study among archaeologists^4^. Increasingly, ancient DNA studies have explored intracommunity social organisation, revealing residency patterns, biological kinship and the social status of past societies^5–10^. For example, closely related individuals have been identified in Late Neolithic and Bronze Age communities from across Europe, often in association with high mitochondrial and low Y chromosomal diversity, indicating female exogamy and a patrilocal social organisation^5–7^. However, few in-depth, site-specific studies such as these have been conducted in this region to date.

**Figure 1:**
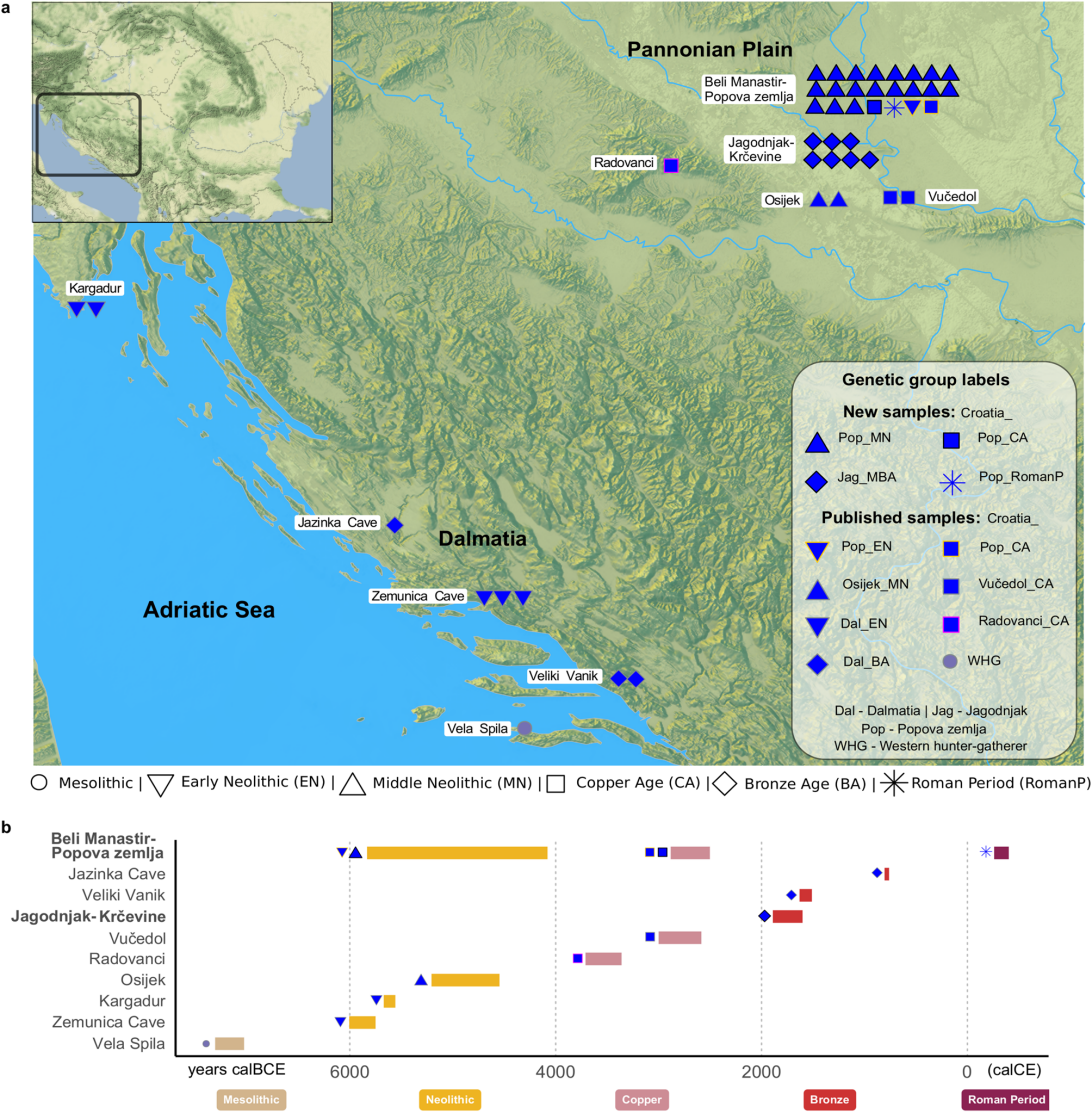
Location and dates of archaeological sites in Croatia. (**a**) Map showing location of archaeological sites for published and newly-reported samples (Table 1, Supplementary Tables S1 and S2). Each point represents a sample, with newly-reported samples outlined in black. Previously-published samples from Popova zemlja are outlined in orange. Genetic group labels include a shortened form of site or region: Pop Popova zemlja, Dal Dalmatia, Jag Jagodnjak. WHG indicates the group label Western Hunter-Gatherer. Shapes correspond to samples of different time periods: EN Early Neolithic, MN Middle Neolithic, BA Bronze Age, CA Copper Age, MBA Middle Bronze Age, RomanP Roman Period. See Methods for further time period labels used for other reference populations. Map made with Mapzen terrain tiles obtained from AWS Public Dataset (https://registry.opendata.aws/terrain-tiles/). European terrain data produced using Copernicus data and information funded by the European Union - EU-DEM layers. Ocean, river and lake data from Natural Earth. Free vector and raster map data @ naturalearthdata.com.Top left inset map of the region, made with map tiles by Stamen Design, under CC BY 3.0. Data by OpenStreetMap, under ODbL. (**b**) Combined radiocarbon and contextual date ranges for samples from present-day Croatia (Table 1, Supplementary Tables S1 and S2, Methods). Site names for newly-reported samples are highlighted in bold. All plots were produced using *R* 3.5.2^102^.

The eastern region of present-day Croatia demarcates the southern edge of the Pannonian Plain (broadly synonymous with the Carpathian Basin), and is intersected by the Danube river, Sava, Drava and other large tributaries that are the site of many prehistoric settlements and formed an important part of communication and exchange networks in this area^11,12^. The emergence of the Neolithic here can be traced to the arrival of the Starčevo culture, which spread from present-day Serbia west and northwards into the Carpathian Basin^13^, while at coastal sites the Early Neolithic was marked by the presence of the Impressed Ware culture from about 6000 BCE^14,15^ (Fig. 1). By 5200 BCE the Starčevo had been superseded by the Sopot culture^16,17^, which practised intramural burial rites, where predominantly children and women were interred under the floors of houses and along walls or other locations within the settlement^18–20^. One important question ancient DNA can help to address is who was selected for such intramural burials, and whether biological kinship played a role. In addition, we can start to unravel whether genetic ancestry and biological kinship are linked to differences in mortuary rites such as body position, burial location within a site, or the distribution of grave goods, which can hint at the existence of different social groups, and could represent ascribed or achieved status of the deceased or the mourners^19,21,22^.

By the Late Neolithic in southeast Europe and southern Transdanubia, a new mortuary practice emerged with the appearance of cemeteries located away from the space of the living^18^. This was accompanied by growing social distinctions among burials^4,23^, signalling an important change in people’s relationship with the dead^18^. The Croatian Copper Age (4500/4300 BCE—2400 BCE) saw the settlement of the Lasinja, Baden, Kostolac and Vučedol cultures, among others, in what is present-day continental Croatia^24–26^, which witnessed the growth of trade networks and more pronounced social hierarchy as seen in the appearance of high status burials^27,28^. The development of more marked social stratification appears to be linked with the growing use of metals in the Bronze Age, which spanned in present-day Croatia from about 2400—800 BCE, and saw a further increase in migrations from the eastern European steppes, the Aegean and Anatolia along with a rise in social ranking^1,26^. One of the numerous Middle Bronze Age cultures to co-exist in the Pannonian Plain was the Transdanubian Encrusted Pottery culture (Supplementary Text S2), which existed in a northern and southern form that extended into present-day eastern Croatia between 2000 and 1500 BCE^12,29^. To date, predominantly cremation burials have been found associated with this culture (Supplementary Text S2), however, now, with the new availability of inhumation burials, we can use ancient DNA to shed light on their genetic and social structure, and use the genetic data to learn more about social status as seen in the distribution of prestige grave goods.

Here we present new genome-wide data for 28 individuals from two sites in present-day eastern Croatia, spanning from the Middle Neolithic to one individual dating to Roman times to investigate what impact the processes of migration and admixture had on genomic variation in this understudied region. Moreover, the presence of divergent mortuary rites at both an intramural burial site and an extramural cemetery from different time periods offers an opportunity to gain valuable insights into biological kinship, demography and social organisation in the context of the changing bio-cultural influences that shaped prehistory in the region.

## Results

### Samples and archaeological background

We screened a total of 54 individuals for whole genome shotgun sequencing (Table 1, Fig. 1a-b, Supplementary Table S1). Of these, we analysed 19 from the Middle Neolithic layer of Beli Manastir-Popova zemlja (abbreviated to Popova zemlja hereafter; Croatia_Pop_MN), which constitutes the largest Sopot culture habitation site to have been excavated in Croatia to date (Supplementary Text S1). Almost half of those excavated were under the age of 16, suggesting high subadult mortality. Two thirds of these were female, while males and females were represented equally among the adults. Most individuals were inhumed with Neolithic burial rites in contracted position along the walls of large pit houses or in other pits within the habitation site, sometimes with ceramic grave goods placed near their heads and other household items. Three of those sampled (POP07, POP09, POP14) (Table 1) were accompanied by a comparatively large number and variety of grave goods consisting of everyday items related to household and economic activities. Another four sampled individuals were deposited mostly in an extended prone or supine position in a channel running along the eastern edge of the site with few grave goods. New radiocarbon dates were also generated for one Copper Age individual (Croatia_Pop_CA) and one Roman period individual (Croatia_Pop_RomanP) from this site (Table 1, Fig. 1a-b, Supplementary Table S1). Approximately 12 km south lies the Middle Bronze Age biritual necropolis of Jagodnjak-Krčevine (abbreviated to Jagodnjak hereafter), attributed to the Transdanubian Encrusted Pottery culture (Supplementary Text S1, Supplementary Text S2). We analysed a further seven inhumations from this site (Croatia_Jag_MBA), which also contains over 30 cremations contextually attributed to the same period (Supplementary Text S1). Inhumations here contain varying degrees of grave good richness ranging from ceramic wares to gold personal ornaments. We co-analysed these new groups with published data from West Eurasian populations, in particular further Middle Neolithic individuals from present-day Croatia (Croatia_Osijek_MN), the Copper Age at Popova zemlja (Croatia_Pop_CA) and the wider region (Croatia_Radovanci_CA; Croatia_Vučedol_CA), and the Dalmatian Bronze Age (Croatia_Dal_BA), as well as groups across different time periods in present-day Hungary and the Balkan Peninsula^2^ (Fig. 1a,b, Supplementary Table S2).

**Table 1:** Summary information for ancient samples first reported in this paper. Capture data and calibrated radiocarbon dates for POP07 and POP14 were previously reported in^2^. See Methods and Supplementary Table S1 for further information about data provided in the table.

Individual ID	Site name, archaeological time period and culture	Date range (BCE contextual; calBCE/calCE 95.4% CI calibrated radiocarbon age)	Population analysis label	Genetic sex	# autosomal SNPs overlapping with 1240 K panel	mtDNA haplotype	Y haplotype
POP02	Beli Manastir-Popova zemlja Middle Neolithic Sopot	4700–4300 BCE	Croatia_Pop_MN	F	855,968	K1a	
POP04				M	884,196	H	J
POP05				F	790,411	K1a5	
POP06				F	944,648	K2b1	
POP07		4790–4558 calBCE		M	861,721	U5b2b	I2a2a
POP08		4700–4300 BCE		F	805,956	U8b1a1	
POP09				F	867,653	K1a4	
POP11				F	865,887	T2b3	
POP12				F	841,209	T2b	
POP13				F	800,065	T2c1d1	
POP14		4763–4536 calBCE		F	913,421	N1a1a1	
POP16		4700–4300 BCE		F	825,473	N1a1a1a2	
POP19				F	878,975	N1a1a1a3	
POP24				M	795,338	K1a1a	I2a2a
POP27				F	837,294	T2b21	
POP30				M	805,808	T2b11	G2a2a
POP33		4603–4071 calBCE		M	710,341	K1a1	G2a2b2a1a1
POP35		4584–4458 calBCE		M	774,710	J2b1a5	C1a2b
POP36		4700–4300 BCE		M	759,163	H	G2a2a
POP39	Beli Manastir-Popova zemlja Copper Age	2859 – 2502 calBCE	Croatia_Pop_CA	F	981,784	HV9	
POP23	Beli Manastir-Popova zemlja Roman Period	260–402 calCE	Croatia_Pop_RomanP	M	962,966	T2f2	R1a1a1b2a2b1
JAG06	Jagodnjak-Krčevine Middle Bronze Age Southern Transdanubian Encrusted Pottery Culture	1800–1600 BCE	Croatia_Jag_MBA	M	846,845	T2b11	G2a2a1a2a2a1 ~
JAG34		1879–1642 calBCE		M	919,781	K2a	G2a2a1a2a2a1 ~
JAG58		1800–1600 BCE		M	762,207	T2b11	G2a2a1a2a2a1 ~
JAG78				M	922,257	U5b1b1a	G2a2a1a2a2a1 ~
JAG82				M	869,661	U2e1a1	G2a2a1a2a2a1 ~
JAG85				F	734,912	K1b1b1	
JAG93				F	807,256	U5a1g	

We conducted all sampling and processing of samples in dedicated ancient DNA laboratories (Methods). We generated whole-genome shotgun data from petrous bone to ~ 1X coverage and aligned fragments to the reference human genome (Methods). We then called pseudo-haploid genotypes using the positions of approximately 1.24 million genome-wide single nucleotide polymorphisms (SNPs) (Methods). As samples were non-UDG treated, we limited analyses to transversion-only SNP sites to mitigate against erroneous genotype calls caused by ancient DNA damage. We confirmed DNA molecules were ancient by observing short read lengths and damage patterns at the ends of reads typical in ancient molecules (Table 1, Supplementary Table S1, Methods). Mitochondrial and nuclear DNA contamination estimates did not exceed 2% for all individuals (Table 1, Supplementary Table S1, Methods). All samples therefore passed quality control measures, and were included for further population genetic analysis. Genetic sexing identified fifteen female and thirteen male individuals (Table 1, Supplementary Fig. S1, Supplementary Table S1). We merged the data from the newly reported individuals with a published worldwide dataset of 1311 present-day individuals genotyped on the Human Origins (HO) array^30^ (Methods), and data from 1102 published ancient individuals (Methods, Supplementary Table S2) after filtering for contaminated samples and first-degree relatives. All passing individuals had at least 700,000 SNPs available for downstream analyses (Table 1).

### Genetic transformations from the Neolithic to Roman times

**Figure 2:**
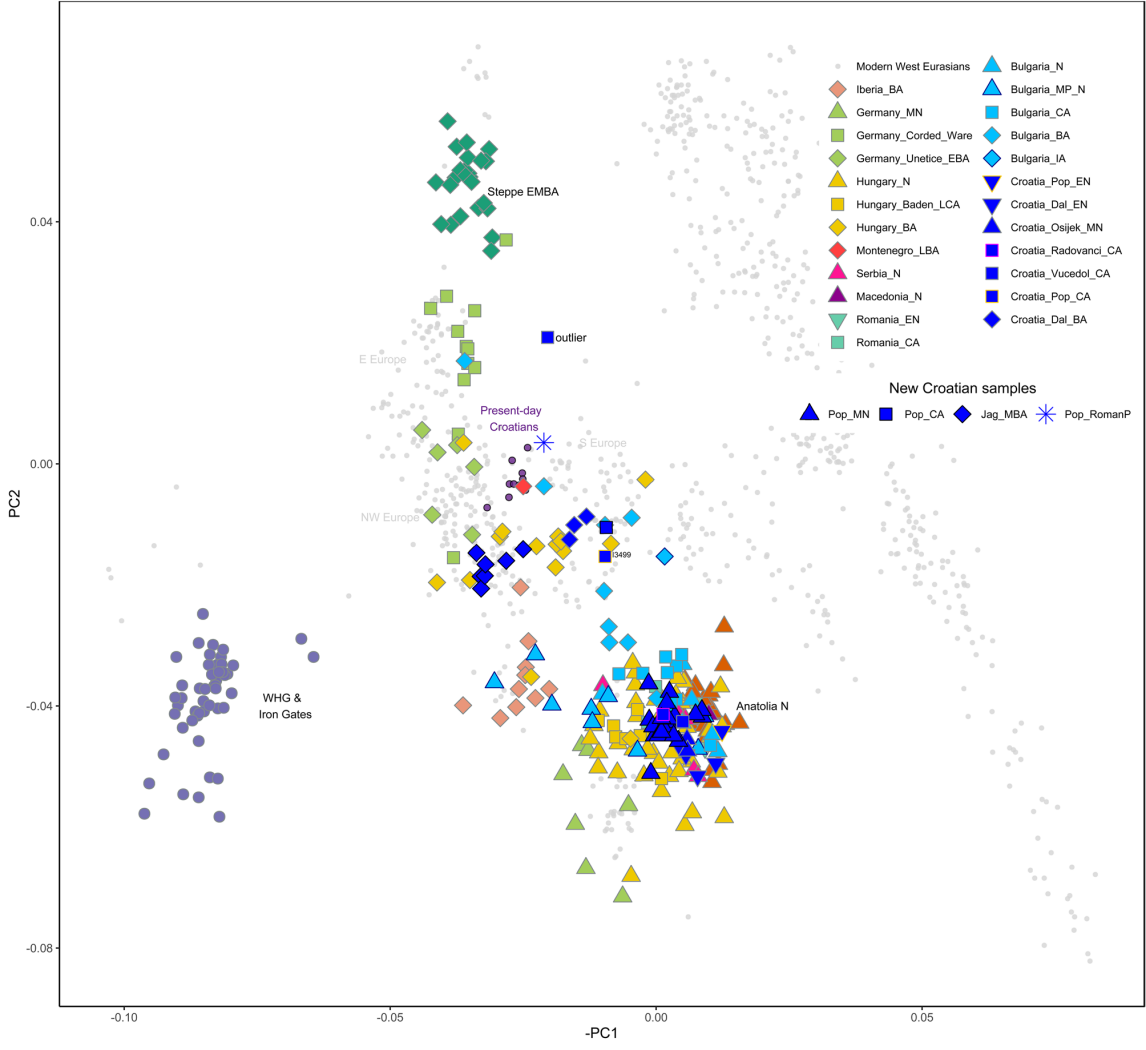
West Eurasian PCA. PCA plot of 59 modern West Eurasian populations with a projection of new individuals from this study and selected published ancient genomes using shrinkmode in smartpca. New samples from this study are indicated with a black outline. Present-day Croatian samples are shown as filled dark purple round points. One previously published individual, Croatia_Vučedol_CA, (I4175, see Supplementary Table S2) is labelled “outlier” in the PCA and was excluded from further analysis based on its position in the PCA, low coverage and lack of radiocarbon date. Plot produced using *R* 3.5.2^102^.

In order to understand the genetic affinities of the samples, we performed principal components analysis (PCA) (Methods) by projecting the new shotgun data and published ancient datasets onto the first two principal components constructed from 920 individuals selected from contemporary West Eurasian populations in the Human Origins (HO) dataset^30^ (Methods, Fig. 2). We also performed model-based clustering analysis in an unsupervised mode with ADMIXTURE (Methods) using 1311 present-day individuals taken from a panel of worldwide populations (Supplementary Fig. S2).

**Figure 3:**
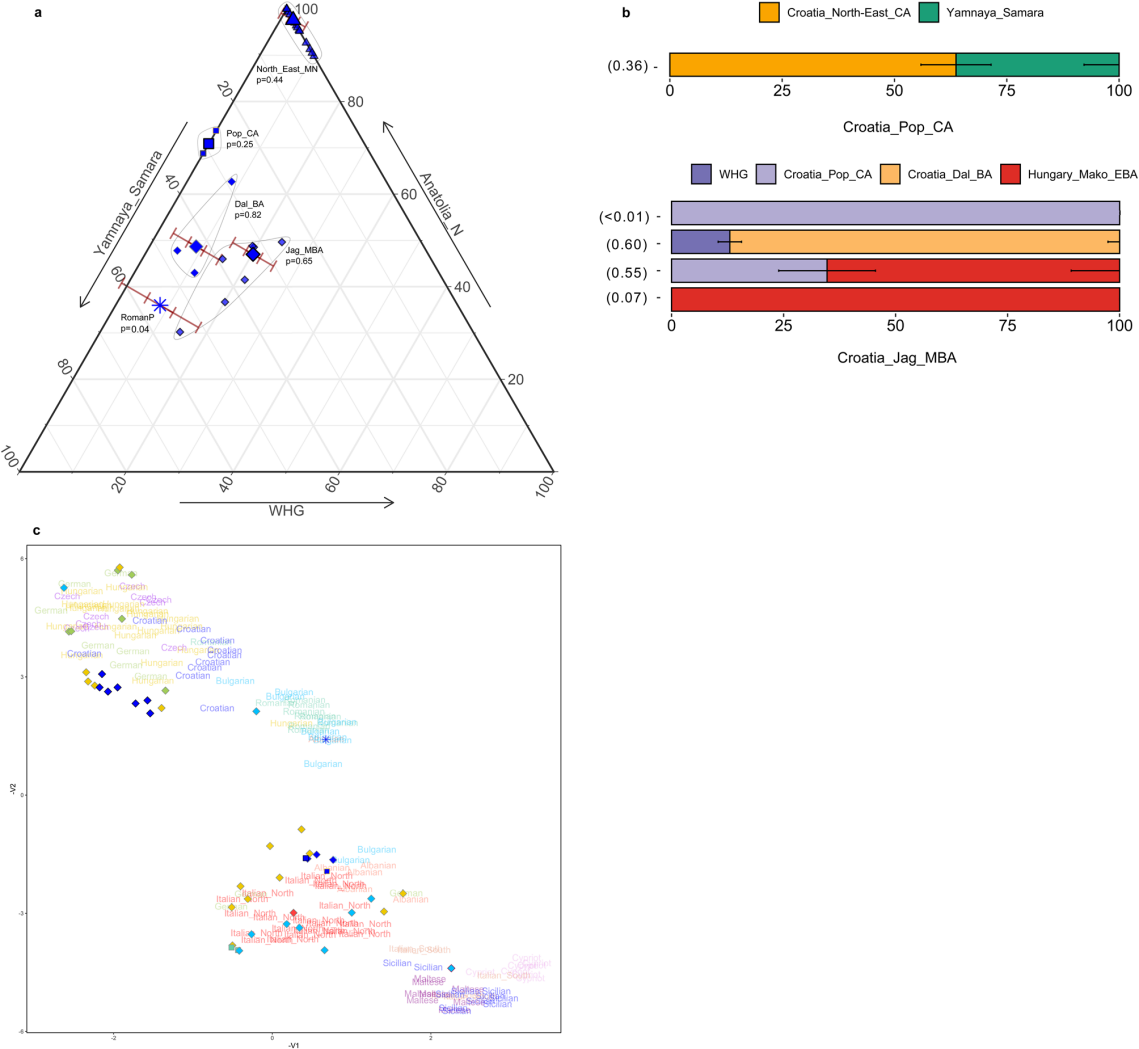
Admixture and genetic affinities in ancient Croatian genomes. (**a**) Distal admixture models obtained with qpAdm for ancient Croatian genomes (Source data in Supplementary Table S4). Smaller points represent individuals and larger points represent population groups with one and three standard error bars indicated for the WHG component. Each population group is labelled with its p-value and the group is encircled for clarity. Croatia_North-East_MN results are for nested two-way models with sources WHG and Anatolia_N, Croatia_Pop_CA is modelled with Anatolia_N and Yamnaya_Samara, and the remainder are three-way models using sources WHG, Anatolia_N and Yamnaya_Samara. **b**) Proximal admixture models for Croatia_Pop_CA and Croatia_Jag_MBA using qpWave and qpAdm (Source data in Supplementary Table S4). Error bars are one standard error in each direction. (**c**) UMAP plot of selected post-Neolithic genomes from southern and central Europe. Present-day populations from the Human Origins dataset are indicated with text; ancient genomes are indicated with points as per PCA symbols in Fig. 2. All plots were produced using *R* 3.5.2^102^.

The newly-reported individuals fall along the European cline in PCA space, extending between Neolithic agriculturalist and Bronze Age pastoralist populations. Croatia_Pop_MN clusters tightly with other southeast and central European Neolithic and Copper Age individuals, including Copper Age Croatians from Radovanci and Vučedol, who were merged for further analysis into Croatia_North-East_CA, and share similar ADMIXTURE profiles that exhibit a major contribution from Anatolia-related ancestry (Anatolia_N) and a small contribution of Western European hunter-gatherer (WHG)-related ancestry (Supplementary Fig. S2). We also merged Croatia_Pop_MN and Croatia_Osijek_MN to form Croatia_North-East_MN for further analysis, before testing shared drift with other ancient and modern West Eurasian populations with outgroup *f*_*3*_-statistics of the form *f*_3_(Croatia_North-East_MN, Test; Mbuti.DG) (Supplementary Fig. S3a-b, Supplementary Table S3, Methods). This group shares most genetic drift with other Neolithic populations from the Balkans and Central Europe, and present-day Sardinians. We then quantified admixture proportions with qpAdm using distal sources of WHG and Anatolia_N to represent Mesolithic hunter-gatherer and Anatolian Neolithic farmer ancestries that are known to have contributed to European genomic diversity (Methods). We were able to model Croatia_North-East_MN as a mixture of 2.4 ± 1% WHG and 97.6 ± 1% Anatolia_N, and even a 100% Anatolia_N model fits the data (p = 0.11), which is congruent with previous studies that show very low WHG introgression in the Balkans and Hungarian Neolithic^3,31^ (Fig. 3a, Supplementary Fig. S4, Supplementary Table S4). Using Iron Gates hunter-gatherers (Iron_Gates_HG) instead of WHG produced very similar results (Supplementary Table S4). Using DATES (Methods), we estimated the timing of this admixture to between 19 and 42 generations before the contextual date of the samples (Supplementary Fig. S5, Supplementary Table S5, Methods), corresponding to the Early Neolithic. This further supports population continuity during the Middle Neolithic, in contrast to Middle Neolithic populations from central and Western Europe which show additional WHG gene flow during this time^31^.

We grouped the new Copper Age individual, POP39, with a previously published cladal individual, I3499 (Supplementary Table S2, Supplementary Table S6), who originates from the same site and time period (Croatia_Pop_CA). This group is shifted further up along PC2 and clusters with three previously reported Bronze Age samples from coastal Dalmatia (Croatia_Dal_BA), falling within the wide distribution of Bulgarian and Hungarian Bronze Age genomes and present-day southern Europeans in PCA space (Fig. 2) suggesting the presence of steppe-related ancestry. Indeed, distal admixture modelling with qpAdm estimates a contribution of 71 ± 8% from Anatolia_N and a further 29 ± 8% from Yamnaya_Samara, representing steppe-related ancestry absent in the Neolithic but found widely among Eurasian Copper and Bronze Age populations. (Fig. 3a, Supplementary Fig. S4, Supplementary Fig. S6, Supplementary Table S4). We obtained a feasible two-way admixture model with the more proximal, broadly contemporaneous pre-steppe group Croatia_North-East_CA (64 ± 8%) and Yamnaya_Samara (36 ± 8%) (Fig. 3b, Supplementary Table S4).

We considered the newly-reported Middle Bronze Age genomes from Jagodnjak (Croatia_Jag_MBA) a single group for further population genetic analysis based on common archaeological context and clustering on the PCA (Fig. 2). We observe a marked shift left along PC1 towards Western and Iron Gates hunter-gatherers, with which it shares the most drift in outgroup *f*_*3*_-statistics (Supplementary Fig. S3, Supplementary Table S3). Distal admixture modelling using sources WHG, Anatolia_N and Yamnaya_Samara confirms a large WHG component in Croatia_Jag_MBA (20 ± 2%), in contrast to Croatia_Pop_CA, and is more than double the fraction estimated for the broadly contemporaneous Dalmatian Bronze Age (Fig. 3a, Supplementary Fig. S4, Supplementary Table S4), also consistent with the significantly positive F4 tests of the form *f*_*4*_(Mbuti.DG, WHG; Croatia_Dal_BA, Croatia_Jag_MBA) (Z = 6.95) (Supplementary Table S7). The Jagodnjak group also harbours slightly greater steppe-related ancestry compared to the preceding Croatia_Pop_CA at 33 ± 5% (see also Supplementary Fig. S6), consistent with previous findings for the Balkan region^2^. Replacing WHG with Iron_Gates_HG harbours comparable results (Supplementary Table S4). This group falls at the left side of the wide distribution of Bronze Age populations from the Carpathian Basin in PCA space, as well as present-day NW European genomes such as French, suggesting an eastward expansion of the Western Bronze Age signature.

To further characterise differential genetic affinities of the Jagodnjak group to the Dalmatian Bronze Age and other genomes, we visualised genetic substructures among post-Neolithic genomes from the region with increased resolution using UMAP and default parameters (Fig. 3c, Methods). While UMAP does not reflect genetic distances in a linear way, clearly defined clusters become apparent, in which Croatia_Pop_CA and Croatia_Dal_BA cluster with ancient genomes from Bulgaria, Montenegro, Romania and some Hungarians together with predominantly present-day Italian_North genomes, pointing to a genetic profile consistent with southern Europe. Testing with qpWave confirmed that Croatia_Pop_CA provides a feasible single source of ancestry for the Dalmatian Bronze Age (Supplementary Table S4). Croatia_Jag_MBA contrastingly falls to the left of present-day genomes from Hungary, Germany, Czech Republic and Croatia, indicating a Central European genetic signature. Other ancient genomes in this cloud also include individuals from the Carpathian Basin belonging to Makó EBA, Vatya MBA, and an LBA individual.

The excess WHG-related ancestry present in Middle Bronze Age Jagodnjak suggests that this group descends from populations harbouring additional WHG-related ancestry that is lacking in the preceding Croatian Copper Age or Dalmatian Bronze Age, consistent with qpAdm modelling (Fig. 3b, Supplementary Table S4). Archaeological evidence points to exchange networks between the Middle Bronze Age communities of eastern Croatia and other cultural groups further north^29^. Based on its date and core distribution in the Carpathian Basin^32^, as well as its clustering with Croatia_Jag_MBA in UMAP and PCA space, we considered Hungary_Makó_EBA the most suitable candidate source of ancestry. This choice is further supported by Hungary_Makó_EBA sharing similar amounts of drift with WHG to Croatia_Jag_MBA (Supplementary Fig. S7, Supplementary Table S3). We indeed obtained feasible models with Hungary_Makó_EBA, either as a two-way model with 35 ± 11% contribution from Croatia_Pop_CA or as a single source (Fig. 3b, Supplementary Table S4).

For Croatia_Jag_MBA we estimated the date of admixture between WHG and Anatolia_N as 41 ± 13 generations before the combined radiocarbon and contextual date of the population (Supplementary Fig. S5, Supplementary Table S5). This is consistent with a calendar date range of 3424—2412 BCE, which overlaps with the Copper Age. We also explored potential sex bias in the inheritance of ancestral components with qpAdm applied separately to autosomes and the X chromosome. While results are consistent with no significant sex bias (-1 < Z-score < 1), the large standard errors in such analyses may hide low or moderate differences between the sexes (Supplementary Table S8, Methods).

### Genetic transformation following the Bronze Age

**Figure 4:**
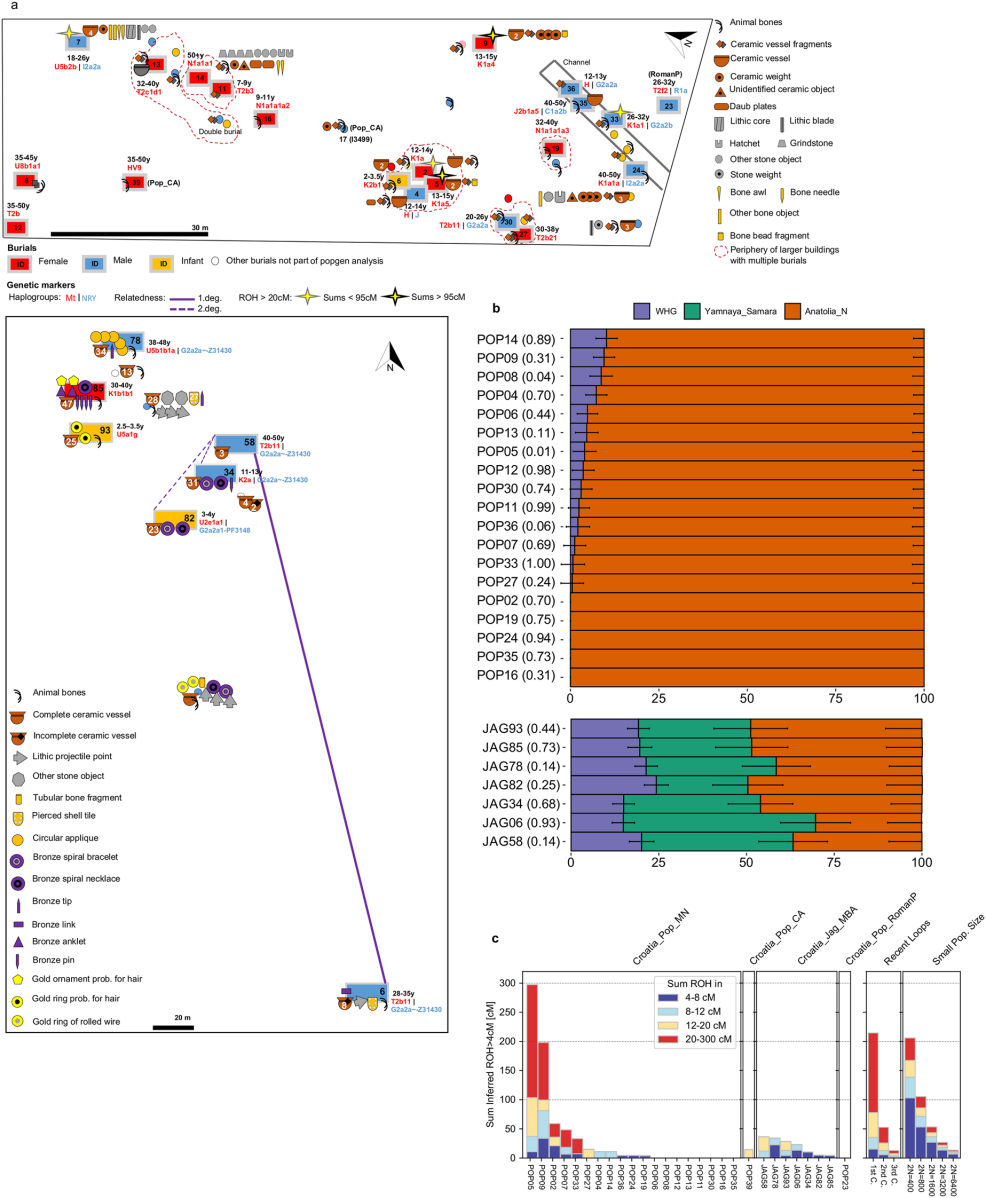
Burials and individual measures of genetic variation. (**a**) Plan of Popova zemlja (top) and Jagodnjak necropolis (bottom) showing inhumations, grave goods and demographic information. Burials with increased translucency indicate these were not sampled due to poor preservation or uncertain context. Burials without a fill colour indicate preservation was too poor for sex estimation. Number of grave good objects exceeding one are indicated. Estimated age at death is indicated as a range in years (y) for individuals included in population genetic analysis. Individuals with an estimated age at death of under 8 years are indicated as infants. (**b**) Stacked bar plots showing distal admixture models calculated with qpAdm for individuals from Croatia_Pop_MN (top) and Croatia_Jag_MBA (bottom), with p-values stated in brackets. Nested two-way models between WHG and Anatolia_N are shown for Croatia_Pop_MN. Three-way admixture models between WHG, Anatolia_N and Yamnaya_Samara are shown for Jagodnjak. Standard error bars are shown as one standard error in each direction (Source data in Supplementary Table S4). Plot produced using *R* 3.5.2.^102^ (**c**) Sums of inferred runs of homozygosity (ROH) greater than 4 cM calculated for each individual with hapROH (Source data in Supplementary Table S10). “Recent loops” and “Small Pop. Size”, generated by hapROH, show the expected distribution of ROH from simulated data for close parental relationships (C = cousin) and small effective population sizes respectively. Plot produced using *R* 3.5.2^102^.

Neither the Jagodnjak nor the Dalmatian Bronze Age groups approximate present-day populations from the region in PCA space, indicating that further significant population changes have since occurred. Our single individual from Popova zemlja, Croatia_Pop_RomanP, provides rare genomic data for Croatia after the Bronze Age^33^. (Table 1, Fig. 4a, Supplementary Table S1). We find this individual clustering with present-day populations of Croatia, Bulgaria and Romania in PCA and UMAP space (Figs. 2, 3c).We investigated this clustering with *f*_4_-statistics and could confirm cladality of this individual with present-day Croatians compared to other ancient and present-day populations in Europe (Supplementary Table S7). We then tested population continuity with qpWave, and found Croatia_RomanP was consistent with forming a genetic clade with present-day Croatians, as well as Bulgarians or Hungarians (p = 0.78 respectively) (Supplementary Table S4). Although based on a single individual who may or may not be representative for the wider population in that time period, this data point indicates that a broadly present-day genetic signature had already formed by Roman times, and any further population turnovers were not as significant as previous ones.

### Intra-population genetic diversity, kinship and demography

We next investigated intra-population genetic heterogeneity and patterns of demography by analyzing individual ancestry, haplotypic diversity, consanguinity and runs of homozygosity (ROH) (Fig. 4a–c).

Individual ancestry modelling with qpAdm confirms high genetic homogeneity among all Middle Neolithic individuals, with a large majority exhibiting either no or low Western hunter-gatherer ancestry introgression at p > 0.01 (given multiple testing) and no significant differences between burial rites (Fig. 4a-b, Supplementary Table S4). Jagodnjak MBA individuals also display unstructured ancestry but a greater degree of heterogeneity particularly in the proportion of steppe ancestry (26–55 ± 10%).

Mitochondrial haplotypes assigned with Haplogrep (Methods) identified high haplotypic diversity in the Neolithic (Table 1, Fig. 4a, Supplementary Table S1). A single individual carries an mtDNA haplogroup associated with European hunter-gatherer populations (U5)^3^, while almost 60% of haplotypes belong to branches K and T2. These, together with clades N1a and J account for most of the variation reported in early Neolithic Starčevo and Linearbandkeramik farming communities of northern Croatia and the adjacent Carpathian Basin^3^, pointing to genetic continuity. Y chromosomal haplogroups assigned with Yleaf (Methods), similarly show a high degree of diversity, with seven males represented by four different haplogroups (Table 1, Fig. 4a, Supplementary Table S1). Two of these, C and I, are found in Mesolithic populations, while G2a is commonly associated with the Neolithic expansion^3^. We also detected high mtDNA haplotypic diversity in Jagodnjak, with two males carrying the same defining mutation for T2b11, while three U subclades and two K subclades, present in Mesolithic populations, are also represented. Y chromosomal haplogroups are restricted to the G2a clade however, four of which belong to the same haplotype G2a2a-Z31430. The fifth individual lacks a read covering the determining mutation and can be assigned the next upstream haplotype. These shared haplotypes indicate relatedness between individuals, which was further explored with genome-wide kinship analysis.

Pairwise genome-wide mismatch rates (Fig. 4a, Supplementary Fig. S8, Supplementary Table S9, Methods) identified JAG58 as a first degree relative of JAG06, and their shared mtDNA and Y chromosomal haplotypes (Table 1, Fig. 4a, Supplementary Table S1) are consistent with the interpretation that these men were full siblings rather than father and son. Additionally, JAG58 is a second degree relative of JAG34 and JAG82. JAG06 and JAG34 also have lowered pairwise mismatch rates indicative of a third-degree or more distant consanguineous relationship, as do JAG78 and JAG93. An adult woman, JAG85, is the only individual not part of any pedigree.

We did not identify first or second degree genetic kin among the Middle Neolithic individuals, however POP05 has a lowered pairwise mismatch rate indicative of a more distant consanguineous relationship with another two subadults (POP02 and POP04), all buried in the same pit house. POP05 also has a lowered pairwise mismatch rate with POP24, an older adult male deposited in the channel. Three of these individuals also share the same maternal K1a haplogroup (Table 1, Fig. 4a, Supplementary Fig. S8, Supplementary Tables S1 and S9). Although high inbreeding coefficients can inflate kinship coefficients, (see below), taken together, this suggests they are part of the same matrilineal pedigree. POP24, deposited in the channel, also appears distantly related to POP02, POP04 and POP07, with whom he shares the same Y chromosomal haplotype I2a2a-M223.

We estimated runs of homozygosity greater than 4 cM (centiMorgans) with hapROH (Methods) to assess levels of inbreeding and infer past mating practices (Fig. 4a and c, Supplementary Fig. S9, Supplementary Table S10). Long ROH > 20 cM indicate recent consanguineous mating, whilst many short runs suggest more distant limitations on effective population size. Eight Middle Neolithic individuals have no ROH, pointing to a lack of inbreeding and thus a large mating pool. Among the remainder however, two individuals, POP05 and POP09, harbour many long ROH > 20 cM that strikingly sum to over 95 cM. This is consistent with them being the offspring of first cousins or equivalent, a rarity in the ancient DNA record^34^. A further three individuals have fewer long ROH > 20 cM indicative of their parents being second cousins or equivalent, while the remainder display ROH pointing to relatedness within the last ten generations. Contrastingly, individuals from Jagodnjak exhibit much lower sums of ROH, with no long runs > 20 cM, and some short runs among all individuals suggestive of more distant relatedness. Infant JAG93 and JAG58 have ROH > 12 cM suggestive of parental relatedness up to five generations ago, although shorter ROH is found in JAG58’s first degree relative JAG06, demonstrating some heterogeneity that is mirrored in their admixture profiles. Copper Age and Roman Period individuals show few to no ROH greater than 4 cM.

Finally we analysed a panel of functional SNPs associated with phenotypic traits under recent selection^35–40^ (Supplementary Table S11, Methods) and found that derived alleles for lighter skin pigmentation (SLC45A2 and SLC24A5) and lighter eye colour (HERC2)^35–37^ are present in individuals from all time periods, consistent with previous findings that point to the derived allele for SLC24A5 rapidly increasing in frequency during the Neolithic as a result of migration^38,39^ . In addition, all individuals carry ancestral alleles for the SNP responsible for lactase persistence into adulthood among Europeans (LCT rs4988235) ^39,40^, suggesting they may not have been able to digest lactose as adults, and is concordant with previous findings that lactose tolerance in Europe remained at low frequency into the Bronze Age^38,39^.

## Discussion

With this study we have extended the spatio-temporal genomic transect of present-day Croatia, shedding light on the genetic history of people living in its diverse ecoregions. The distribution of genomes along the European cline in PCA space attests to the importance of this region as a contact zone for migrating peoples across continental Europe over millennia. The Sopot community of Popova zemlja exhibits﻿ genetic continuity from Early Neolithic Starčevo predecessors, supporting previous findings^3^, and displays low levels of WHG-related ancestry that persist into the Copper Age, mirroring the genetic profile of some other contemporaneous populations of the Carpathian Basin^31^. In addition, the Copper Age individuals from Popova zemlja represent an early presence of people with steppe-associated ancestry in this region, who would have co-existed with pre-steppe Copper Age individuals only 60 km away at Vučedol^2^.

In the Bronze Age we again observe two genetically distinct yet concurrent ancestries in different ecoregions. Two Dalmatian individuals associated with the Cetina culture^2^ are broadly contemporaneous with the latest contextual date for Jagodnjak, yet carry ancestry similar to Copper Age Popova zemlja. This profile persists in a third Dalmatian individual who postdates the genetically distinct Jagodnjak individuals by almost a thousand years^2^. The shared genetic affinities between individuals of Jagodnjak and the contemporaneous Vatya culture further north, distinguished by high WHG-related ancestry, supports archaeological evidence for close interaction and exchange networks between various groups in the Carpathian Basin and the southern Transdanubian Encrusted Pottery communities in present-day eastern Croatia, whose ceramic wares have been found in neighbouring Vatya communities and other contemporaneous groups along the Danube in the Carpathian Basin^1,29^. Moreover, their shared affinities are congruent with archaeological evidence for a common immediate predecessor, widely accepted to be the Late EBA Kisapostag culture, which itself was partly founded upon the widely distributed Makó-Kosihy-Čaka cultural complex^24,29^. These insights thus contribute to long-standing debates about the relationships between the different Middle Bronze Age cultural units that co-existed along the Danube and the Carpathian Basin, and reveal genetic affinities between populations of present-day eastern Croatia and the adjacent Carpathian Basin over multiple time periods.

Beyond these spatio-temporal relationships, we have gained valuable insights into the demography and social organisation of these communities. The unstructured and homogeneous ancestry across the Middle Neolithic site, together with high haplotypic diversity and low or no signs of inbreeding in many unrelated individuals is consistent with this community being part of a large, stable and exogamous population, supporting archaeological evidence for high population density in this region^3,4^. Within this context however, we detect a few individuals scattered across the site who exhibit very close parental relatedness. Four of the five belong to the same mtDNA haplogroup clade K1a, while two of these, POP02 and POP05, also have elevated kinship coefficients and are buried next to each other in the largest pit house. Taken together, it is possible these individuals were contemporaneous, and we could be witnessing an example of occasional close relative unions within the same matrilineal pedigree. No other detectable differences distinguish these individuals from others buried at the site in terms of their genetic profile or mortuary rites, suggesting this was a socially acceptable alternative mating choice.

This first genome-wide study of an intramural burial site in this region also reveals no first or second degree relatives, with only a few individuals like POP05 and POP24 sharing more distant consanguineous relationships as well as mtDNA haplogroups. While this could indicate the presence of some distant matrilineal relationships, which also interestingly suggests connections between those deposited in the channel (POP24) and others buried in the main intramural site, close biological kinship does not form the foundation for selection to be buried here, questioning suggestions that those buried together in buildings represent extended families^4^. This may not have been the only form of burial practice however, with biological kin buried elsewhere^18,20^. The high representation of children, in particular girls, as well as burials of neonates, likely signifies age and sex selection and an ascribed status based around the community’s belief systems, for which various explanations have been put forth regarding similar Neolithic intramural sites in the region^20,41^. For example, buildings have been associated with ideas of matrilineality and ancestor worship, and as spaces linked to the reproduction and continuity of society, where the burials within would have provided protection and prosperity ^20,41^. In addition, we do not detect genetic structuring in relation to mortuary practices here. In terms of grave goods, the expression of interpersonal distinctions is limited, with the majority of burials containing few objects such as ceramic vessels or fragments. However a small number of individuals (POP07, POP09, POP14) from different age and sex categories have abundant grave good items associated with everyday activity that appear to signal some limited social differentiation based on social and economic status. The youngest of these individuals is estimated to have been 13–15 years old, thus all were old enough to participate in adult work, and it is therefore difficult to know whether they achieved or inherited their status. Finally, flexed burials in the habitation area versus predominantly male, extended depositions in the channel also do not correlate with detected genetic ancestry. While the reason for the different channel depositions cannot be known, the presence of mixed body positions such as found here has been recorded at other Sopot sites in the Pannonian Basin^19,22,42^, where it has been suggested that it may represent social groupings adhering to different mortuary customs. Considering the highly exogamous genetic profile at this site within a high population density region, we cannot exclude this as one possible explanation. Indeed, Neolithic mortuary rites and population composition have been shown to vary greatly^19,22,43^, therefore more localised studies such as this will help further our understanding of the diversity of this phenomenon.

The recovery of Middle Bronze Age inhumations furnished with varying grave good richness at Jagodnjak offers a rare opportunity to investigate the genetic profile of a culture that to date has been more often associated with cremation burial rites^1,29,44^. Similar to other Transdanubian Encrusted Pottery burial sites (Supplementary Text S2) and Bronze Age burials in Europe more broadly, here we find grave goods comprising prestige items that indicate increased social differentiation compared to the Neolithic^24,27,28^. Firstly, we observe differences in funerary treatment between closely related kin, JAG06 and JAG58 (Fig. 4a). JAG06’s burial contains a lithic arrow tip, a bronze item and a perforated shell, together with a number of ceramics, while JAG58’s grave is larger and deeper yet composed of only a very small number of ceramics and post-cranial bones. This apparent difference in mortuary rites could reflect differential status that was acquired through their lifetimes, or birth order may have been a factor in inheritance of wealth or status. There are indications however of secondary manipulation of these burials, thus the possibility that some grave goods and skeletal elements were disturbed following primary deposition invites a cautious interpretation. One of the most richly furnished graves in terms of number and variety of grave goods belongs to the adult woman JAG85. Comprising the highest number of ceramic vessels as well as gold hair ornaments and numerous other bronze items, her grave furnishings may reflect a high social status that she acquired, or inherited through her birth family or affinal relationships. Bronze items of personal adornment such as pins and jewellery are found in both male and female graves at the site which likely demonstrates the status or wealth of the person or their family^21^, while arrow tips are only found in adult male graves, indicating these individuals’ different status in society. We also observe rich infant graves, JAG82 and JAG93, with the latter containing gold hair rings. These individuals were too young to have achieved wealth or status themselves, suggesting vertical inheritance, likely from family, which has been observed at other Transdanubian Encrusted Pottery contexts (Supplementary Text S2) as well as other archaeological cultures in Bronze Age Germany and Serbia^6,10^.

The relatively high mitochondrial haplotype diversity and very low Y chromosomal diversity among related males suggests the inhumed individuals belonged to a community characterised by female exogamy and adherence to a patrilocal social organisation,also observed at other Late Neolithic and Bronze Age cemeteries in Europe^5–8^. The short runs of homozygosity carried by these individuals is consistent with populations that harbour some distant shared ancestry, possibly due to past constraints on effective population size. Overall, the burials of two brothers, a number of more distant male kin and one unrelated, high status adult woman, together with another five unsampled male inhumations, indicates the possible presence of sex-biased burial practices^8,9^ among a male pedigree observing patrilocality and female exogamy. Many nearby Bronze Age sites recorded in present-day eastern Croatia, including those of the Transdanubian Encrusted Pottery culture^29^, could have been plausible sources of local population interaction, indicating a complex tapestry of Middle Bronze Age communities living on the fringes of the Pannonian Plain.

## Materials and methods

### Radiocarbon dating

We sampled the petrous part of the temporal bone of samples POP23 and POP39 and obtained calibrated radiocarbon dates from the Oxford Radiocarbon Accelerator Unit for POP23 (OxA-378000; OxA-37801, ORAU) and POP39 (OxA-37999, ORAU) using IntCal13 calibration curve^45^ and OxCal version 4.3.2^46^. We also report the following radiocarbon dates for individuals included in this study: from the Ruđer Bošković Institute for POP33 (Z-5732, IRB) with IntCal13 calibration curve^45^ and OxCal version 4.2.4^47^, from the Penn State AMS ^14^C Facility for POP35 (PSUAMS-4444, PSU) with IntCal13 calibration curve^45^ and OxCal version 4.3.2^48^; from University of California Irvine Keck-Carbon Cycle AMS facility for JAG34 (UCIAMS-233509, UCI KCCAMS) with IntCal20 calibration curve^49^ and OxCal version 4.4^48^ (Table 1, Fig. 1b, Supplementary Table S1).

### Group labels

Group labels generally follow the format “Country_SiteName/Region_TimePeriod”, with the site name contracted to a short form, for example Croatia_Pop_MN; Pop Popova zemlja, Jag Jagodnjak, Dal Dalmatia. Time periods are labelled as: BA Bronze Age, CA Copper Age, EBA Early Bronze Age, EN Early Neolithic, IA Iron Age, LBA Late Bronze Age, LCA Late Copper Age, MBA Middle Bronze Age, MN Middle Neolithic, N Neolithic and RomanP Roman Period.

### Sample processing

We processed all samples in dedicated ancient DNA laboratories at the University College Dublin, Ireland. Petrous bones were UV irradiated for 10 to 15 min on each side followed by light sandblasting of the outer surface to remove loose debris. The cochlea was then excavated from the petrous bone using a sandblaster, and UV irradiated on each side for 10 min before it was finely powdered in a mixer-mill (Retsch).

### DNA extraction

DNA was extracted from about 50 to 70 mg of bone powder following a modified silica column based method optimised for ancient DNA samples^50^. In a pre-digestion step aimed at reducing contamination^51^, the bone powders were digested for one hour at 56 °C without rotation in 1 ml of extraction buffer containing 0.45 M EDTA and 0.25 mg/ml Proteinase K. The bone powder was spun down to a pellet by centrifugation and re-suspended in fresh extraction buffer. Samples were digested for one hour at 56 °C followed by 18 h at 37 °C with rotation. Samples were centrifuged at 13,000 rpm, and the 1 ml supernatant added to the reservoir of a Roche High Pure extender assembly tube containing 13 ml of binding buffer. Binding buffer consisted of 5 M Guanidine Hydrochloride, 40% isopropanol, 90 mM sodium acetate and 0.05% Tween-20. Tubes were centrifuged for four minutes at 1500 rcf, the spin column detached and placed in a collection tube and dry spun at 6000 rpm for one minute to remove remaining binding buffer. After placing the spin column in a fresh collection tube, 650 μl of PE wash buffer was added, centrifuged for one minute at 6000 rpm and the flow-through discarded. This step was repeated once followed by dry spinning at 13,000 rpm to remove remaining wash buffer. The column was placed in a clean Eppendorf tube and the sample eluted with 25 μl TET which had been incubated at 37–56 °C. Samples were then incubated at 37 °C for ten minutes followed by centrifugation for 30 s at maximum speed. The elution step was repeated, resulting in a total volume of 50 μl DNA extract. One negative control containing only extraction buffer was processed for every seven samples.

### Library preparation and sequencing

Non-UDG-treated, double-stranded libraries were constructed following^52^. Blunt-end repair was carried out by adding NEBNext End- Repair module (New England Biolabs) to 12.5 μl of each DNA extract, which was incubated at 25 °C for 15 min, followed by 12 °C for 5 min. Samples were then incubated at 25 °C for 30 min for adapter ligation with T4 DNA Ligase (ThermoFisher Scientific). Adapter fill-in was performed with Bst Polymerase (New England Biolabs) with an incubation at 37 °C for 30 min and 80 °C for 20 min to inactivate the enzyme. Purification steps following blunt-end repair and adapter ligation was performed with the MinElute PCR purification kit from Qiagen. A negative control was processed for every seven samples, and a final library volume of 40 μl obtained.

Single indexing PCR was performed by adding a unique seven-base-pair index to 3 μl of each library using Accuprime Pfx Supermix (Life Technology) and IS4 primer to a total reaction volume of 25 μl. The PCR temperature profile consisted of initial denaturation at 95ºC for five minutes, a further 15 s of denaturation at 95ºC, twelve cycles of annealing at 60 ºC for 30 s, elongation at 68 ºC for 30 s and a final extension at 68 ºC for five minutes. A negative PCR control was included with every batch. The PCR amplification and subsequent clean-up steps were performed in a separate lab in a different part of the building from the clean labs. MinElute PCR purification kit spin columns were used for purification of amplified libraries following Qiagen instructions. Quantification of amplified product was performed using Qubit 2.0 Fluorometer (Thermo Fischer Scientific) and Agilent 2100 Bioanalyser DNA 1000 assay. Single-end shotgun sequencing was performed by pooling samples in equimolar amounts onto an Illumina NextSeq500 platform using 75-cycle kits for 1 × 76 cycles and 1 × 7 cycles for de-multiplexing.

### Sequence processing

Adapter sequences were trimmed from reads using Cutadapt (version 1.15)^53^ discarding reads under 17 bp (-m 17) and allowing an overlap of 1 bp between the read and adapter (-O 1). Reads were then mapped to the UCSC genome browser human reference hg19 (GRCh37) to produce BAM files with BWA aln/samse (version 0.7.15-r1140)^54^, replacing the mitochondrial genome with the revised Cambridge Reference Sequence (rCRS, Gen bank accession no. NC_012920.1)^55^. Seed length was disabled (–l 1000) and the default number of differences (-n 0.04) and minimum Phred scale mapping quality of 30 (-q 30) were used. PCR duplicates were removed with SAMtools rmdup (v.0.1.19-96b5f2294a)^56^.

### Genotyping

Sites overlapping the ~ 1240 k SNP capture array were used to generate a pileup file for each individual using SAMtools mpileup (version 1.3)^56^ with the quality flags –q30, -Q30 and –B. This file was used to genotype individuals whereby a single base call was chosen at random from each SNP site to produce pseudo-haploid calls with transversion SNPs only using the flag –t SkipTransitions in pileupCaller (https://github.com/stschiff/sequenceTools) in order to remove variants affected by post-mortem damage in non-UDG treated samples. A second genotype dataset was produced that included all SNPs for use in functional SNP and ROH analyses (see below) (Supplementary Table S1).

### Datasets

We used mergeit (version 2450) from the package ADMIXTOOLS^57^, to merge the new genotype data to reference datasets^2,30,31,35,38,39,75–101^, Supplementary Table S2) containing 1250 ancient individua l s genotyped at 1,233,013 SNP sites, of which 140,159–192,648 transversion-only SNP positions are covered by the newly-reported individuals (Supplementary Table S1). This was also merged with diploid genotypes of 26 present-day individuals^58–61^, and, for tests involving present-day comparisons, a panel of worldwide present-day populations containing 1311 individuals genotyped at 597,573 nuclear SNP positions on the Affymetrix Human Origins (HO) array^30^, of which 66,880–94,317 transversion-only SNP positions are covered by the newly-reported individuals (Supplementary Table S1).

### Phenotypic SNPs

We produced a pileup from BAM files using SAMtools mpileup (version 1.3)^56^ with a minimum mapping and base quality of 30 (-Q 30, -q 30) and –B to turn off base alignment quality for five phenotypically informative SNPs that are included in the 1240 K panel. This included lactase persistence^39,40^ and skin and eye pigmentation^35,36,37^. Numbers of reads supporting each allele are reported in Supplementary Table S11.

### Sex determination

Coverage on the autosomal and sex chromosomes was calculated using a script available at https://github.com/TCLamnidis/Sex.DetERRmine to determine genetic sex of each individual with standard errors. Males should have an x-rate of 0.5 and y-rate of 0.5. Females should have an x-rate of one and y-rate of zero (Table 1, Supplementary Fig. S1, Supplementary Table S1).

### Ancient DNA authentication

The presence of deamination damage patterns at the terminal bases of reads, characteristic of ancient DNA, was verified using mapDamage (version 2.0.8)^62^ (Supplementary Table S1).

### X chromosome contamination in males

As males have only one copy of the X chromosome, we measured contamination in males by estimating polymorphism on the X chromosome using ANGSD (version 0.910)^63^. Results based on new Method1 are reported for a minimum of 200 SNPs on the X chromosome that are covered at least twice (Table 1, Supplementary Table S1).

### mtDNA contamination

Estimates of contamination based on comparison of the mitochondrial genome with a database of potential present-day contaminant human mtDNA sequences were obtained with Schmutzi^64^. To do this, we used EAGER (version 1.92.55)^65^ to remap all reads for each individual to the mitochondrial rCRS (Gen bank accession no. NC_012920.1)^55^ with CircularMapper (version 1.93.4)^65^, filtering on a minimum mapping quality of 30 and removing duplicates. Schmutzi confidence intervals are given as est.high and est.low (Table 1, Supplementary Table S1).

### Mitochondrial haplogroup assignment

We used Schmutzi^64^ to reconstruct consensus mtDNA sequences for each individual from the remapped reads, which we then imported into Haplogrep2^66^ (https://haplogrep.i-med.ac.at/) for automated mitochondrial haplogroup assignment based on phylotree mtDNA tree build 17 (http://www.phylotree.org/) (Table 1, Fig. 4a, Supplementary Table S1). We manually checked aligned mtDNA sequences for individuals possessing the same haplogroup, which revealed that the pair of first degree relatives, JAG58 and JAG06, possessed identical mutations for the T2b branch. Variants at unreliable polyC stretch positions were disregarded: 518, 309.1C(C), 315.1C, AC indels at 515–522, 16093C, 16182C, 16183C, 16,193.1C(C) and 16,519.

### Y chromosomal haplogroup assignment

We used Yleaf (version 1.0)^67^ to infer the Y chromosomal haplogroup in males in an automated way based on haplogroup-defining SNP positions in the ISOGG 2016 nomenclature. We filtered results on derived alleles and transversion-only SNPs, and the most downstream haplogroup was selected (Table 1, Fig. 4a, Supplementary Table S1). Haplogroups were also inferred manually using SAMtools mpileup –q30 –Q30 with concordant results (version 1.3)^56^. We used Integrative Genomics Viewer (Broad Institute)^68^ to visually inspected reads in order to verify if defining variants were in the middle or at the end of a read to assess reliability, and to confirm mutations among individuals with shared haplotypes. Four of the Jagodnjak males share the same mutations (G2a2a-Z31430) while no reads covered the defining position for this haplotype in the fifth male, JAG82.

### Kinship analysis

Consanguinity up to two degrees of relatedness was assessed by calculating pairwise mismatch rates from autosomal pseudo-haploid genotype data filtered on transversion-only SNP sites from the 1240 K SNP panel, using pMMRCalculator (https://github.com/TCLamnidis/pMMRCalculator) and READ^69^ with default parameters. READ provides an upper and lower Z score to help assess the certainty of the results, with the upper Z score indicating the distance to a lower degree of relationship, and a lower Z score indicating the distance to a higher degree of relationship. There was a minimum overlap of 92,000 SNPs between pairs of individuals, and both methods produced comparable results (Fig. 4a, Supplementary Fig. S8, Supplementary Tables S1 and S9), although READ produces slightly lower mismatch rates than pMMRCalculator, meaning individuals are estimated to be slightly more closely related than pMMRCalculator estimates. READ’s binned approach with sliding windows may contribute to this discrepancy. A pair of first degree relatives is expected to have a pairwise mismatch rate that is halfway between the baseline for unrelated and identical individuals. Coefficients of relatedness can be somewhat inflated for individuals with high inbreeding coefficients however. No relatedness was found between POP39 and a previously published individual I3499 from the same site and similar radiocarbon date. Where first degree relatives were identified, one individual from the pair was excluded from population-wide analyses, in this case JAG06. The same analyses were performed on a genotype dataset that included all SNP sites in order to assess the effects of damage on kinship estimates (Supplementary Fig. S8). Transversion-only genotypes shift the pairwise mismatch rates downwards, which means individuals are estimated as more closely related than when using all SNPs. This data is produced from non-UDG-treated DNA libraries, therefore this could indicate that ancient DNA damage can lead to an under-estimate of true relatedness.

### Runs of homozygosity

Runs of Homozygosity (ROH) greater than four centimorgans (cM) were identified with the Python package hapROH^34^ (https://test.pypi.org/project/hapsburg/) using default parameters for the dataset containing all SNPs. A global dataset of 5008 haplotypes were used as a reference panel taken from the 1000 Genomes Project. The total sum ROH is reported for each length category of 4-8 cM, 8-12 cM, 12-20 cM and > 20 cM (Fig. 4a,c, Supplementary Table S10).

### Principle components analysis

We used smartpca (version 16,000) in the EIGENSOFT package (version 6.0.1)^70^ with a set of 59 present-day west Eurasian populations from the Human Origins dataset^30^ to construct the first two principal components, and projected the ancient genomes with options lsqproject:YES, shrinkmode:YES and outliermode:2 (Fig. 2). Present-day populations in the HO dataset used for computing the principal components included: Abkhasian, Adygei, Albanian, Armenian, Balkar, Basque, BedouinA, BedouinB, Belarusian, Bulgarian, Canary_Islander, Chechen, Chuvash, Croatian, Cypriot, Czech, Druze, English, Estonian, Finnish, French, Georgian, Greek, Hungarian, Icelandic, Iranian, Italian_North, Italian_South, Jew_Ashkenazi, Jew_Georgian, Jew_Iranian, Jew_Iraqi, Jew_Libyan, Jew_Moroccan, Jew_Tunisian, Jew_Turkish, Jew_Yemenite, Jordanian, Kumyk, Lebanese, Lezgin, Lithuanian, Maltese, Mordovian, North_Ossetian, Norwegian, Orcadian, Palestinian, Polish, Russian, Sardinian, Saudi, Scottish, Sicilian, Spanish, Spanish_North, Syrian, Turkish, Ukrainian.

### UMAP

UMAP was run with the R package *umap* (version 0.2.3.1)^71^ using default parameters (Fig. 3c). Input was provided from the first ten principal components computed by PCA for 128 individuals from 13 present-day HO popula tions (Albanian, Romanian, Bulgarian, Cypriot, Greek, Italian_North, Italian_South, Maltese, Sicilian, Czech, Hungarian, German, Croatian) and 47 ancient individuals (Germany_Untetice_EBA, Hungary_BA, Montenegro_LBA, Romania_CA, Bulgaria_BA, Bulgaria_IA, Croatia_Dal_BA and the newly-sequenced individuals.

### ADMIXTURE analysis

We performed unsupervised admixture analysis with ADMIXTURE (version 1.3.0)^72^ (Supplementary Fig. S2) on 2,361 ancient and present-day individuals (see Datasets section in Methods) by first using PLINK (version 1.90b5.3)^73^ to remove variants that had a minor allele frequency below 0.01, and to prune the dataset for strong linkage disequilibrium with parameters –indep-pairwise 200 25 0.4. We then ran five replicates for K4 to K17 with a random seed and cross-validation (Supplementary Fig. S2), and the highest likelihood replicate was chosen.

### *f*-statistics

We used a set of packages in ADMIXTOOLS^57^ for performing *f*-based statistics. Outgroup *f*_*3-*_statistics was calculated with qp3Pop (version 435) (Supplementary Fig. S3﻿a-b, Supplementary Fig. S7, Supplementary Table S3), qpDstat (version 751) was used to calculate *f*_*4*_-statistics with the option f4Mode: YES (Supplementary Fig. S6, Supplementary Table S7), and qpWave (version 410) and qpAdm (version 810) were used with option allsnps: YES for estimating mixture proportions (Fig. 3a-b, Fig. 4b, Supplementary Fig. S4, Supplementary Table S4). The option Chr: 23 was added to qpAdm for computing results based on the X chromosome in analyses testing for sex-bias (Supplementary Table S8). Following the method outlined in^2^, we calculated a Z score for each ancestry component to measure the difference in admixture proportions between the autosomes and X chromosome, where a positive Z score indicates more admixture on the autosomes and therefore male-biased ancestry. Mbuti.DG was used as an outgroup for all statistics. For qpAdm, right populations included Mbuti.DG, Ust_Ishim_HG_published.DG, Ethiopia_4500BP.SG, Russia_MA1_HG.SG, Italy_Villabruna, Papuan.DG, Onge.DG, Han.DG. qpWave was used to check the outgroup populations could successfully distinguish the ancestries present in the sources. Rather than identifying the specific source populations and admixture events that occurred, qpAdm models help to ascertain the type ancestry that would have contributed to the gene pool of the target population via admixture.

### Admixture dating

We used DATES (https://github.com/priyamoorjani/DATES)^74^ to estimate the age of past population admixture events between two source populations by inferring time since mixture from the average size of ancestry blocks, assuming a generation time of 29 years (Supplementary Table S5). Decay curves are reported in Supplementary Fig. S5. Estimates can contain some noise due to later admixture events, and this model does not take into account multiple admixture events or admixture of already admixed populations.

## Supplementary Information

Supplementary Information 1.

Supplementary Information 2.

## Data Availability

All source data needed to evaluate the conclusions in the paper are present in the paper and/or the Supplementary Materials. Data generated for the ancient individuals first reported in this study is available through the European Nucleotide Archive (ENA) (http://www.ebi.ac.uk/ena) under study accession number PRJEB46357.
