# Supplementary material for: Reconstructing genetic histories and social organisation in Neolithic and Bronze Age Croatia
Source: Sci Rep. 2021 Aug 18;11:16729. doi: 10.1038/s41598-021-94932-9 (PMC8373892; doi:10.1038/s41598-021-94932-9)
Supplement: Supplementary file 1 — Supplementary Information 1. [file 41598_2021_94932_MOESM1_ESM.pdf]

Supplementary Information

Reconstructing genetic histories and social organisation in Neolithic and Bronze Age Croatia

Suzanne Freilich, Harald Ringbauer, Dženi Los, Mario Novak, Dinko Tresić Pavičić, Stephan Schiffels, Ron Pinhasi

Supplementary Text S1 Archaeological information and description of individuals included in this study

Beli Manastir-Popova zemlja, Osijek-Baranja County, Croatia

Following the discovery of archaeological structures belonging to multiple periods from the Early Neolithic to the Roman period, rescue excavations during the construction of a highway in 2014 and 2015, led by D. Los from Kaducej Ltd, recovered a very rich concentration of artefacts[1,2]. Nearly 47,000 pottery fragments, bone objects, stone tools and animal bones were excavated and assigned to the Neolithic context, attesting to a large prehistoric population density. Potsherds belonging to the Neolithic Starčevo, Sopot and Lengyel cultures were recovered, as well as a few from the Copper Age Baden and Vučedol cultures, and two Roman period brick kilns. The whole archaeological site is 550x68 m (45.010 m²) to a depth of approximately 80 cm. The prehistoric settlement on the north side of the site covers more than 15.500 m² (230x68 m).

Most of the Neolithic site consists of large pit houses, with larger ones approximately 100-200m² and 60-90 cm deep. Other structures included fireplaces and stoves, and many channels, probably for drainage of living space. Most burials were found at the bottom, and some on the top layer, of the larger pit houses, indicating the inhumation was performed while the building was still in use

and part of the inhabitants' everyday lives. Burials were always found along the periphery of the structures. Three of the largest ones contained five or six graves usually at the bottom of the structure, suggesting they were the oldest. Further inhumations were recovered from the bottom of other burial pits located near the pit houses, and interpreted as waste pits or secondary purpose pits of unknown use. Most burials were in contracted position on their left or right side in various orientations, and some had ceramic vessels placed by their head. There is variability in burial orientations but a preference for SW-NE. A channel running along the eastern edge of the site and still in use by village inhabitants during Neolithic times, contained the remains of a further seven individuals, mostly males in an unarranged extended supine or prone position with no addition of grave goods except for some animal bones. The channel depositions lack osteological signs of violent death[3,4]. This constitutes the largest number of Neolithic burials found at a single site in Baranja.

Neolithic burials

- **Grave number 02 (POP02)**

A well-preserved skeleton of a female 12-14yrs old in a SW-NE orientation with head facing south and in a foetal position.

- **Grave number 04 (POP04)**

A well-preserved skeleton of a male 12-14yrs old in SW-NE orientation lying on his right side in foetal position.

- **Grave number 05 (POP05)**

A well-preserved skeleton of a female 13-15yrs old in SW-NE orientation, flexed position lying on her right side with hands pressed against torso.

- **Grave number 06 (POP06)**

A poorly preserved skeleton of a female infant 2-3.5yrs old with head oriented to the south, facing the right side, and a S-N body orientation.

- **Grave number 07 (POP07)**

Skeleton of an adult male 18-26yrs old, laying on his back in flexed position towards his left side with right arm bent over torso and placed on upper left arm, in a NE-SW orientation and head facing north.

- **Grave number 08 (POP08)**

A poorly preserved skeleton of a female 35-45yrs old lying in a SW-NE orientation, flexed position on her right side with head facing to the right, and arms pressed against the torso with hands under her head.

- **Grave number 09 (POP09)**

A poorly preserved skeleton of a female 13-15yrs old lying on her left side in an E-W orientation and flexed position with arms pressed against the torso and hands under her head.

- **Grave number 11 (POP11)**

A partially-preserved skeleton of a female 7-9yrs old lying on her left side in flexed position in E-W orientation with head facing east.

- **Grave number 12 (POP12)**

A well-preserved skeleton of a female 35-50yrs old, orientation W-E lying in flexed position on her back with head facing the right side. The right arm was bent towards the head and the left arm bent across the torso.

- **Grave number 13 (POP13)**

A well-preserved skeleton of a female 32-40yrs old in NE-SW orientation and face turned to the left lying on her left side in a foetal position with arms pressed against the torso and hands under her head.

- **Grave number 14 (POP14)**

A partially-preserved skeleton of a female 50+yrs old in a SE-NW orientation lying on her back in flexed position with head facing the left side. Her right arm was bent over the torso and left arm stretched straight down. She was found on the top layer of backfill with traces of burning belonging to the burial. This woman had severe wear of teeth biting surfaces, wedge-shaped vertebrae and lifetime fractures caused by excessive load of her spine.

- **Grave number 16 (POP16)**

A poorly-preserved skeleton of a female, 9-11yrs old in an E-W orientation lying in foetal position on her left side and hands under her head.

- **Grave number 19 (POP19)**

A poorly-preserved skeleton of a female 32-40yrs old lying in W-E orientation on her right side with head facing right. Legs were only slightly flexed and arms bent up to the face.

- **Grave number 24 (POP24)**

A poorly-preserved skeleton of a male 40-50yrs old at the bottom of a channel that was part of a large canal along the eastern edge of the habitation site. Lying on his back with legs outstretched in an extended supine position in a SW-NE orientation and head tilted back.

- **Grave number 27 (POP27)**

A partially-preserved skeleton of a female 30-38yrs old in a W-E orientation lying in a flexed position on her abdomen with head turned to the right side. Left arm bent and pressed against torso with hand against her cheek and right arm below torso. Legs bent at knees to the left side.

- **Grave number 30 (POP30)**

A partially-preserved skeleton of a male 20-26 yrs old lying in a W-E orientation on his left side with face to the ground and thigh bones indicating he was buried in a flexed position.

- **Grave number 33 (POP33)**

A well-preserved skeleton of a male, 26-32yrs old lying in an extended prone position in a NE-SW orientation with head facing to the ground in the channel. The right arm extends down the torso and the left arm is bent at the elbow. Legs are slightly extended with lower leg bones missing due to damage from recent pipe works.

- **Grave number 35 (POP35)**

A partially-preserved skeleton of a male 40-50yrs old lying in a SW-NE orientation in the channel in an extended prone position with head turned to the right.

- **Grave number 36 (POP36)**

A well-preserved skeleton of a male, 12-13yrs old lying in a NE-SW orientation in the channel, lying on his back in a flexed position with legs bent to the left. Left arm is bent up towards the head and right arm stretched over the torso also raised to his head.

Copper Age

- **Grave number 39 (POP39)**

Skeleton of a female 35-50yrs old. A new radiocarbon date was obtained for this individual from ORAU, 2859-2502 calBCE (4095±28 BP, OxA-37999) which places it in the Croatian Copper Age.

Roman Period

- **Grave number 23 (POP23)**

A well-preserved skeleton of a male, 26-32yrs old in an E-W orientation lying on his left side with head turned to the left, in a flexed position. His left arm is bent towards the head and right arm is laid over his torso. Notable bone pathologies included an oval, shallow lifelong healed fracture in the middle of his frontal bone, and a lifelong healed fracture of the seventh left rib, where a small remodelled callus is present. A new radiocarbon date was obtained for this

individual from ORAU, 260-402 calCE (1692±18 BP, combined [(1714±25 BP, OxA-38000), (1670±25 BP, OxA-38001)] which dates to the Roman period. (Two radiocarbon dates were produced by ORAU as part of their internal quality control process.)

Jagodnjak-Krčevine, Osijek-Baranja county, Croatia

Rescue excavations took place at the site of Jagodnjak-Krčevine in eastern Croatia in 2014 and 2015, led by D. Tresić Pavičić from Kaducej Ltd. The site lies about 25km north of the city of Osijek, and contains finds from the Neolithic, Bronze Age, Late Iron Age, Roman and Early Medieval periods. In the part of the gravesite excavated, a biritual necropolis was discovered with 11 inhumations and over 30 cremations belonging to the end of the Early to the Middle Bronze Age Transdanubian Encrusted Pottery Culture, contextually dated to between 1800-1600 BCE. All the recovered cremations were furnished with ceramic vessels, cremated and non-cremated animal remains, and more than 150 metal, mostly bronze, objects. Most of the remains were very fragmented and one quarter of the cremations were double burials that included remains of at least one child. Two thirds of subadults were below five years old, pointing to high subadult mortality, and most adults belong to the younger adult age group. All individuals showed evidence of pathological changes most of which indicated subadult stress, and evidence of degenerative osteoarthritis points to intense physical activity in young adults[5-7]. The evidence suggests bodies were most likely cremated on funeral pyres. Among the inhumation burials in this study, there is a preference for NE-SW burial orientation among adults with head facing SE, body in flexed position on left side, while the two infants are oriented E-W with head facing south. A comprehensive analysis of the graves and grave goods inventory from this site is ongoing and subject to minor modification.

- **Grave number 6 (JAG06)**

A skeleton of a 28-35-year-old male in flexed position in NE-SW orientation lying on left side with head facing SE.

- **Grave number 34 (JAG34)**

A well-preserved skeleton of a 11-13 year-old male lying on his left side in flexed position in a NE-SW orientation with head facing SE. Some post-mortem cortical damage was present due to erosion and weathering. Green stains from metal oxidation were present on the left shoulder, the left ribs, and the right forearm. A trepanation was identified on the frontal bone of his cranium, which is first described in[8].

- **Grave number 58 (JAG58)**

A skeleton of a 40-50-year-old male with only the skull and three other bones preserved. The skull was pointing north with the head facing west.

- **Grave number 78 (JAG78)**

A skeleton of a 38-48-year-old male buried in a NE-SW orientation, lying on his left side with head facing SE. No lower body bones were present so body position could not be determined. Arms were extended forward.

- **Grave number 82 (JAG82)**

A skeleton of a 3-4-year-old male buried in flexed position in E-W orientation lying on his left side, with head facing south.

- **Grave number 85 (JAG85)**

A skeleton of a 30-40-year-old female burial in flexed position on her left side in a NE-SW orientation, with her head facing SE.

- **Grave number 93 (JAG93)**

A skeleton of a 2.5-3.5-year-old female infant found in flexed position lying on her left side in E-W orientation, with head facing south.

Supplementary Text S2 The Southern Transdanubian Encrusted Pottery Culture of the Middle Bronze Age

The following text provides contextual information about the Transdanubian Encrusted Pottery Culture, based on[9] where it has been described in detail.

Distribution

The Transdanubian Encrusted Pottery Culture (TEPC) spanned about 400 years between 2000 - 1600 BCE. It is associated with the alluvial plains near rivers and the lower foothills in western Hungary, however its distribution also extends into eastern Croatia, Slovakia and Romania. Following an early transitional phase, a northern and southern form developed with their own styles of ceramic production. The southern Transdanubian group (STEPC) existed between the Sió and Kapos Rivers, stretching between the Mecsek Hills and the Danube until the Drava River. It also existed across the Drava, at the region of the Danube– Drava confluence. The STEPC inhabited sub-Mediterranean oak forest vegetation zone while the northern Transdanubian Encrusted Pottery Culture (NTEPC) inhabited a sub-Mediterranean and mixed Central European forested zone. At least 26 STEPC sites have been recorded in NE Croatia (Slavonia and Baranja) but these have been largely stray finds and remain unpublished. More notable sites recorded in Slavonia and Baranja include Batina, where a bronze hoard was found, a settlement site with occupation through all phases of the culture at Beli Manastir, and a settlement and burial site occupied through all phases at Karanac. The small number of burials in TEPC cemeteries suggest small-sized, single-layered settlements with rivers separating the two. More rare are hilltop, fortified and cave settlements. Many more sites belonging to the younger phase than the older period points to increasing population density in the Middle Bronze Age.

Chronology and links to other cultural complexes

The Transdanubian Encrusted Pottery Culture (TEPC) and contemporaneous Litzenkeramik developed from the preceding Corded Ware culture. The Late EBA Kisapostag culture is regarded as the immediate predecessor to both the TEPC and the Vatya culture along the Danube. The TEPC can be divided into phases as follows:

Phase 1: Late Kisapostag–Early Encrusted Pottery phase (transitional phase), circa 2000BCE

Phase 2: Older Encrusted Pottery phase

Phase 3: Younger Encrusted Pottery phase

Late phase: Proto Szeremle phase dating to Koszider period, ending circa 1600 BCE

The end of the TEPC is marked by the development of the Szeremle culture and rise of the Tumulus cultures of the Carpathian Basin. It is thought that late TEPC populations may have mixed with late Vatya, Vatin and Maros populations due to intensive exchange networks, leading to the development of the Szeremle culture.

Material culture and exchange networks

Following phase 1, the NTEPC and STEPC developed different methods of ceramic manufacture and decorative styles. The discovery of bronze and gold hoards, ornaments and tools from burials also attests to the TEPC's production of distinctive metalwork. One bronze hoard is known from eastern Croatia at the site of Batina. The northern and southern TEPC groups were connected through trade and/or migration, and locally produced and imported early and late phase TEPC-ware has been found in neighbouring Vatya sites as well as other cultural complexes further north along the Danube including tell sites. During phase 1, the TEPC exchanged pottery for bronze and gold objects from the tell cultures. In the transitional and early phase of the TEPC, imported metal wares were discovered in a high status burial from Balatonakali. In the younger phase, STEPC

wares from the Drava are found as far as the northern and eastern edges of the Great Hungarian Plain and believed to be prestige items. During phase 3 in Croatia, northern and southern TEPC imports are found in mixed assemblages with Litzenkeramik ware. Litzenkeramik populations also lived in Slavonia and Baranja, and an increase in relations is seen towards the late phase of the TEPC. While in the early phases sporadic connections are observable with neighbouring cultures, later on a lot more pottery appears as far as the Czech-German and Transylvanian Ore mountains, suggesting strong inter-regional exchange networks in the second part of the MBA. One Kórós axe traces to the Cetina culture which indicates importation from the eastern Adriatic coast too.

Burial rites

Small, family-based communities are considered characteristic of the period, with up to 50-60 burials, although more recently larger cemeteries are being uncovered too. A change in burial rites has been observed over time, which could be due to chronological factors, mixing of populations or related to the display of different social status. In the STEPC, no evidence has been found for a sudden change in rites due to the arrival of a new population. Rather, it appears to be due to a gradual change of the Kisapostag population, which was characterised by biritual inhumations and cremations. Then in the transitional early phase, inhumations become rare. Only one inhumation was discovered at Balatonakali and at Vörs (NTEPC), and at Bonyhád in the southern group, dated to 2100-1948 BCE. Inhumations became rare after the early phase, when cremation became the dominant form of burial rite. In the NTEPC, mixed rites of urn and scattered cremation tradition were found in both the older and younger phase, but in STEPC, the younger phase was almost exclusively scattered cremation burials, with the exception of Bonyhád. Rare younger phase inhumations were found at the NTEPC site of Balatongyörök, Veszprém and Vörs.

Grave goods and social status

The alternative practice of inhumation was accompanied by grave goods as with cremations. Different burial rites could represent social differences, for example grave good richness is linked to social status as well as gender and age. Females are found to have more metal ornaments than men, and metal tools are more often associated with male burials. Symbols connected to gender have also been identified on pottery. The largest number of pots recorded in STEPC burials is about 35 pieces, with a couple of exceptions that are likely special child burials who were found with over 100 vessels. Children and mature adults have been found to have more grave goods than younger or middle aged adults, albeit not everywhere. Children were also found to often have different ceramic forms and miniatures. Objects such as dress ornaments and bronze jewellery have been found in all age groups, suggesting vertical stratification of society, that is, the inheritance by children of social status from their parents or family. During the Late Kisapostag-Early Encrusted Pottery phase, one inhumation was found in addition to cremations at the site of Balatonakali which was given special treatment as inferred by the rich bronze assemblage and a gold hair ring that accompanied it, suggesting high rank. Another at Vörs also received special treatment. Special grave goods like gold ornaments occur sparingly, suggesting a local elite.

Supplementary Information references

1. Los, D. Results of the archaeological excavations of the site AN 2 Beli Manastir–Popova Zemlja. *Annales Instituti Archaeologici.* **16,** 90-102, Institute of Archaeology, Zagreb (2020).

2. Roguljić, I. O. Trial archaeological excavations at the Beli Manastir–Popova zemlja site (AN 2). *Annales Instituti Archaeologici* **10,** 29–33, Institute of Archaeology, Zagreb, (2014).

3. Novak, M. *Bioarchaeological Analysis of Human Bone Remains from Site Beli Manastir-Popova zemlja (AN2)*. Expert Report BA1 - 05/17, Zagreb, Croatia (2017).

4. Novak, M., Los, D, and Janković, I. Life and Death during the Neolithic at the Edge of the Pannonian Plain: Bioarchaeology of Human Skeletal Remains from Beli Manastir-Popova zemlja, Croatia. Paper presented at the 20th Annual Conference of the British Association of Biological Anthropology and Osteoarchaeology, Cranfield, United Kingdom. (14-16 September 2018).

5. Novak, M., Pavičić, T. D. & Janković, I. Rich grave goods, bad health? The preliminary results of the bioarchaeological study of the Middle Bronze Age cremation burials from Jagodnjak, Eastern Croatia. Presented at the 88th Annual Meeting of the American Association of Physical Anthropologists, Cleveland, OH (27-30 March 2019).

6. Novak, M. *Bioarchaeological Analysis of Human Bone Remains from Site Jagodnjak-Krčevine (AN7)*. Expert Report BA1 - 02/17. Zagreb, Croatia (2017).

7. Novak, M. & Pavičić, D. T. Living and Dying during the Bronze Age in the Pannonian Basin - Mortuary Practices and Health Conditions in the Community of Jagodnjak between 2000 and 1500 BCE. Presented at Prehistoric Communities along the Danube, Osijek, Croatia. (28-30 Nov 2019).

8. Carić, M. *et al.* Something old, something new: (re)analysis and interpretation of three Bronze Age trepanations from Croatia. *Anthropologie*. **LVIII,** 39–51 (2020).

9. Kiss, V. *Middle Bronze Age encrusted pottery in western Hungary.* (Archaeolingua **27,** Budapest, 2012).

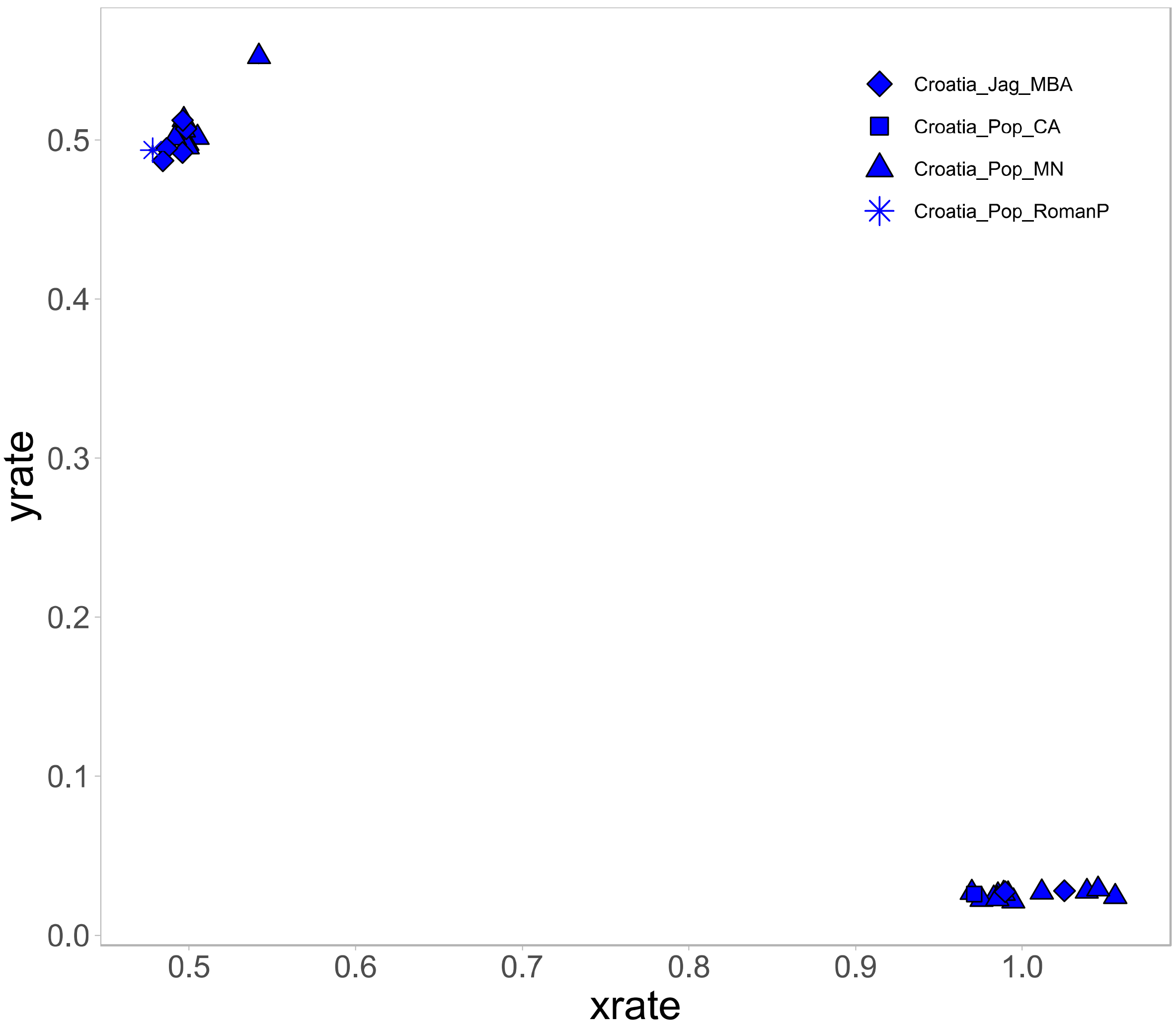

Supplementary Figure S1. Sex determination. Genetic sex determination calculated with https://github.com/TCLamnidis/Sex.DetERRmine showing proportion of reads that map to the x and y chromosomes. An x rate of 1.0 indicates an individual is genetically female, and an x and y rate of 0.5 respectively indicates male genetic sex.

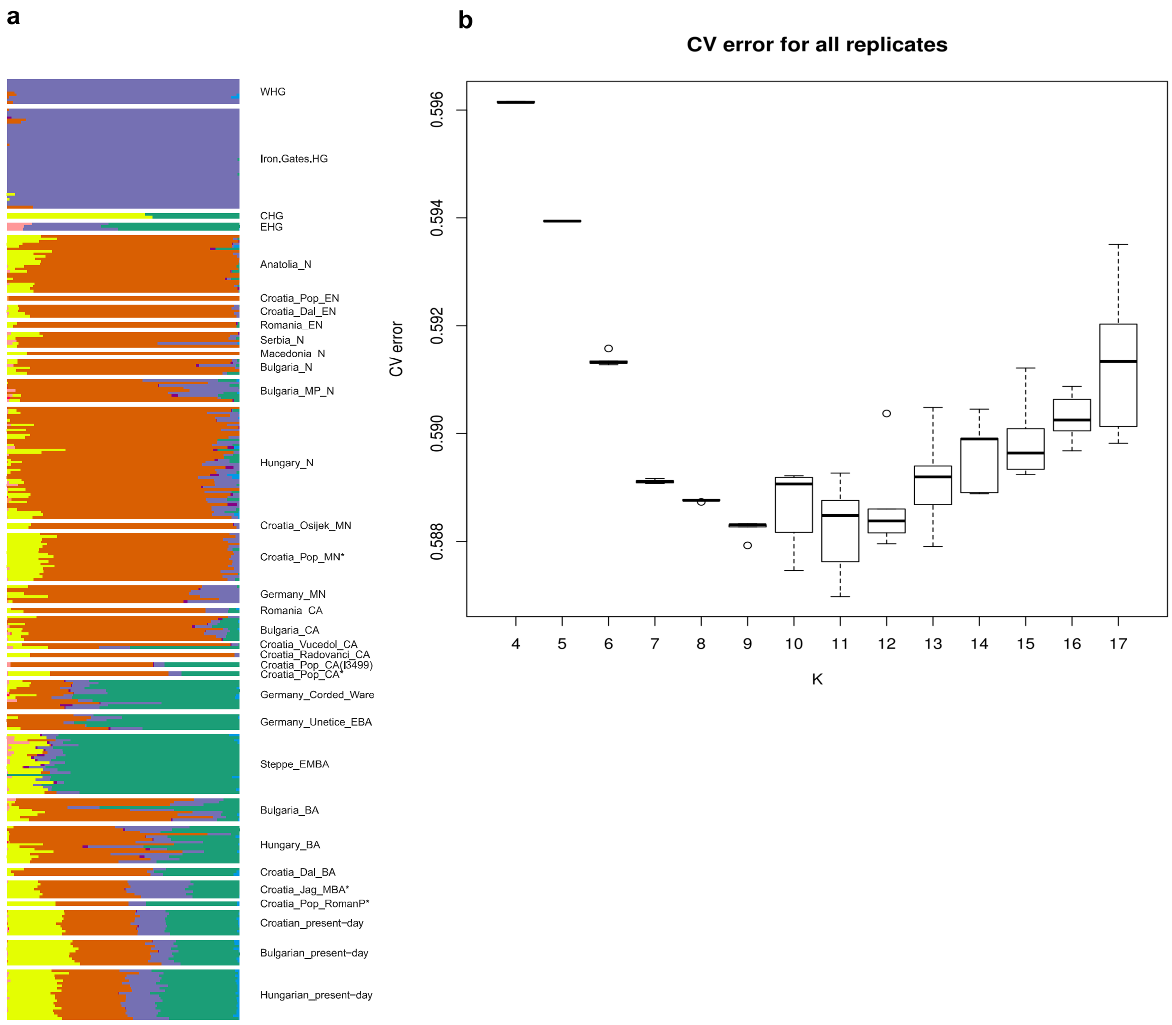

Supplementary Figure S2. Unsupervised ADMIXTURE. (**a**) Plot of ADMIXTURE results (K=9) showing selected Balkan and West Eurasian populations. New individuals reported in this study are indicated with an asterisk. Single bars have been widened for better visibility. (**b**) Cross-validation errors for ADMIXTURE k values from 4 to17 of ancient samples and present-day worldwide populations from the Human Origins dataset. The median value is represented by a solid line, and rectangles indicate the first and third quartile. Whiskers go from the first and third quartile to the minimum or maximum value, and dots indicate outliers.

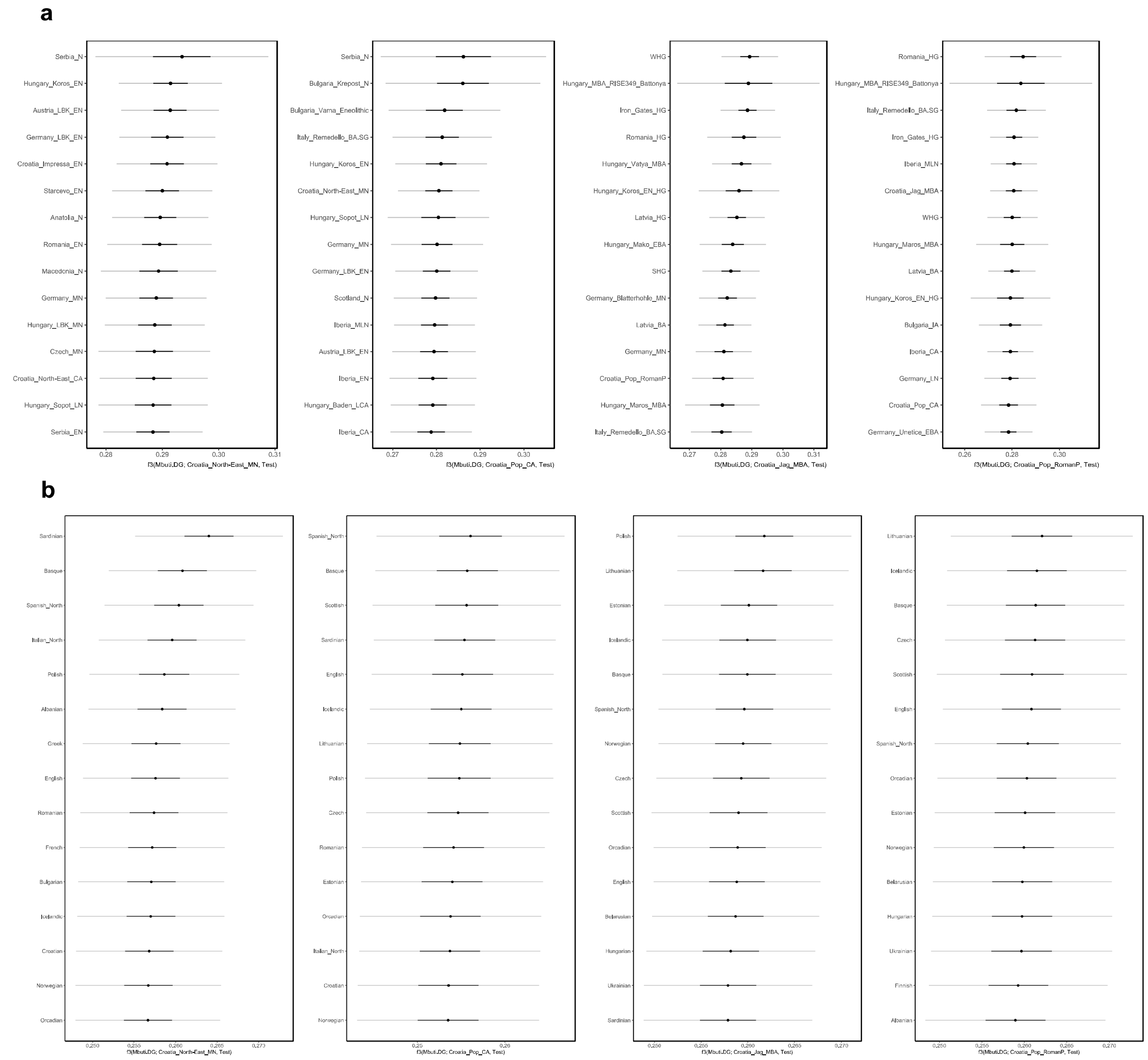

Supplementary Figure S3. Shared genetic drift. Plots showing **(a)** 15 ancient and **(b)** 15 present-day groups from the Human Origins dataset sharing the most genetic drift with the groups from present-day eastern Croatia, using a minimum of 10,000 overlapping SNPs, as measured by the *f*-statistic *f*3(Mbuti.DG; Croatian group, ancient/present-day Test). Black and grey bars indicate one and three standard errors respectively. (Based on data in Supplementary Table S3.)

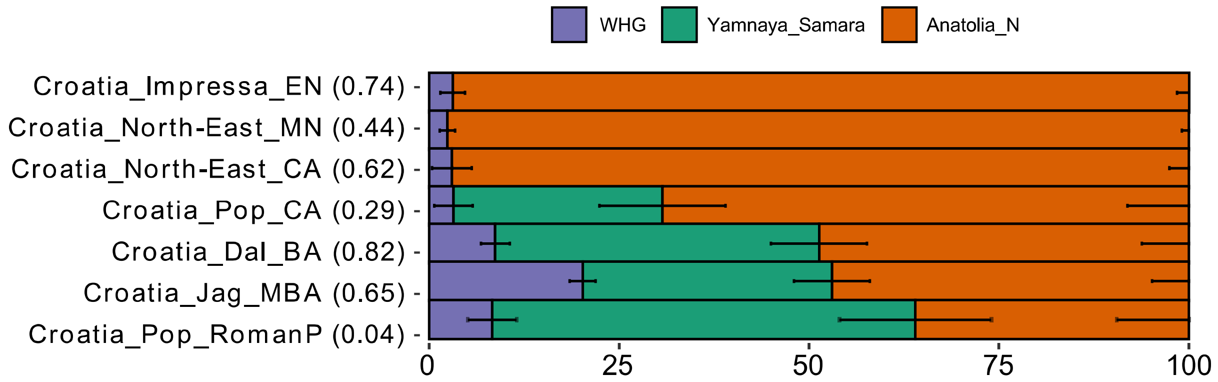

Supplementary Figure S4. Distal admixture models for populations from present-day Croatia. Admixture fractions of published and new groups from present-day Croatia estimated with qpAdm using two and three-way admixture models with three sources WHG, Yamnaya_Samara and Anatolia_N (data in Supplementary Table S4; see here also for alternative working models). Error bars indicate one standard error in each direction. P-values are indicated next to the group name in brackets.

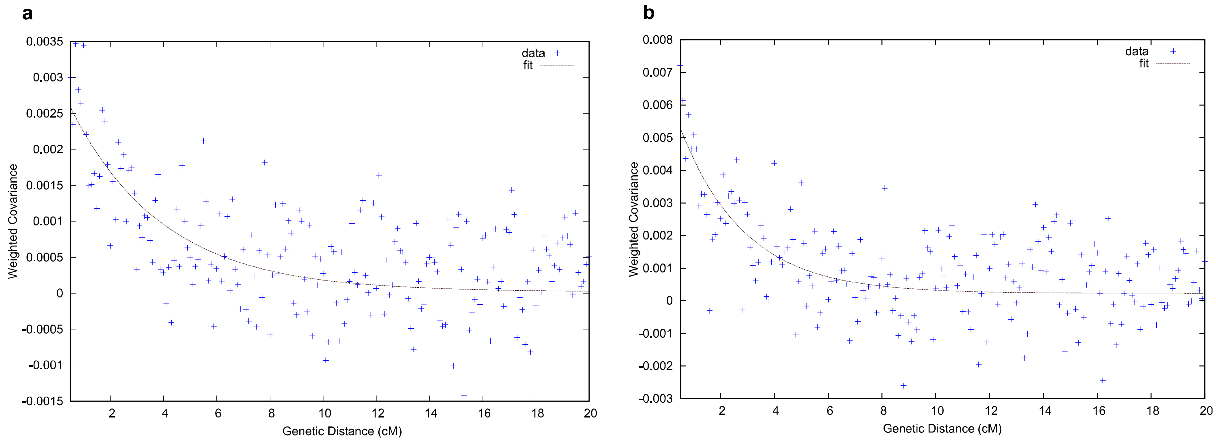

Supplementary Figure S5. DATES decay curves. The decay of ancestry covariance estimated by DATES for WHG and Anatolia_N in (**a**) Croatia_North-East_MN and (**b**) Croatia_Jag_MBA.

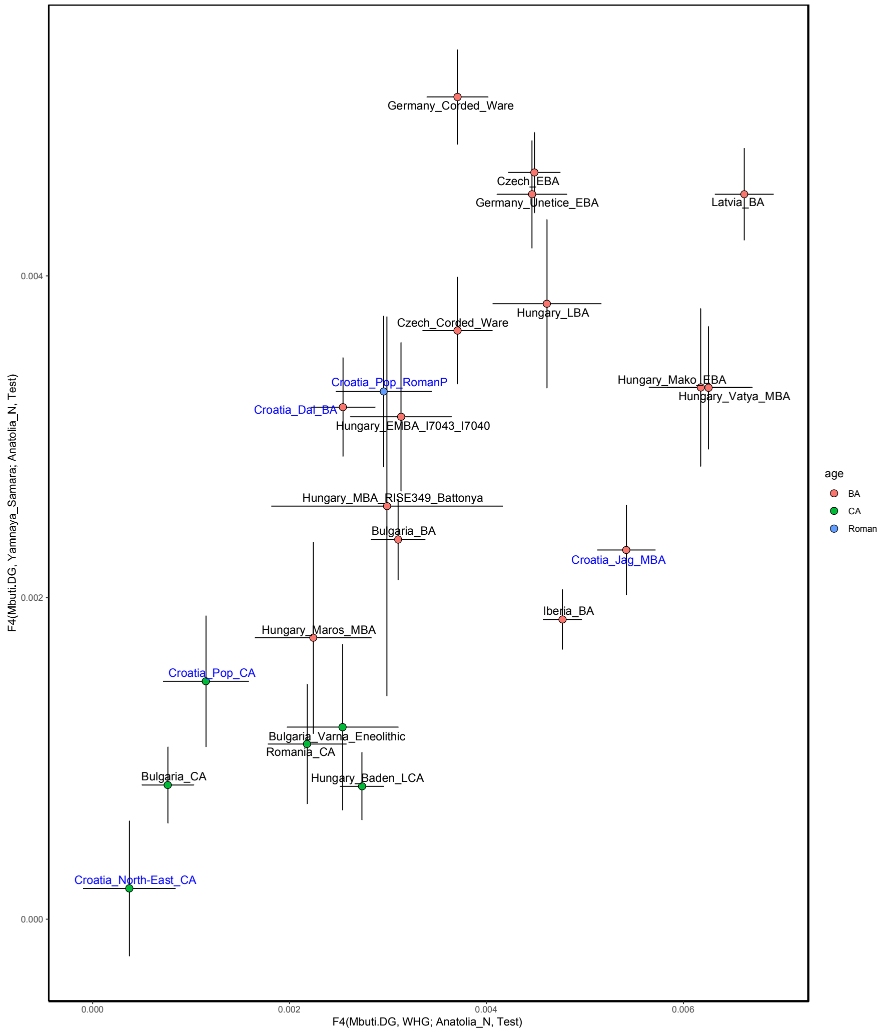

Supplementary Figure S6. Genetic affinities of ancient populations to WHG and Yamnaya_Samara ancestry. An f_4-statistics scatter plot showing differential affinities of selected post-Neolithic ancient Eurasian populations to WHG and Yamnaya_Samara ancestry (data in Supplementary Table S7). The results for the test f_4(Mbuti.DG, WHG; Anatolia_N, Test) are plotted on the x-axis against the results for the test f_4(Mbuti.DG, Yamnaya_Samara; Anatolia_N, Test) on the y-axis with one standard error bar indicated for each axis. Points are coloured according to time periods BA (Bronze Age), CA (Copper Age) and Roman period. Groups from present-day Croatia are highlighted with blue text. Groups further to the right along the x-axis infers increased genetic affinity to WHG, while groups further up along the y-axis infers increased genetic affinity to Yamnaya_Samara.

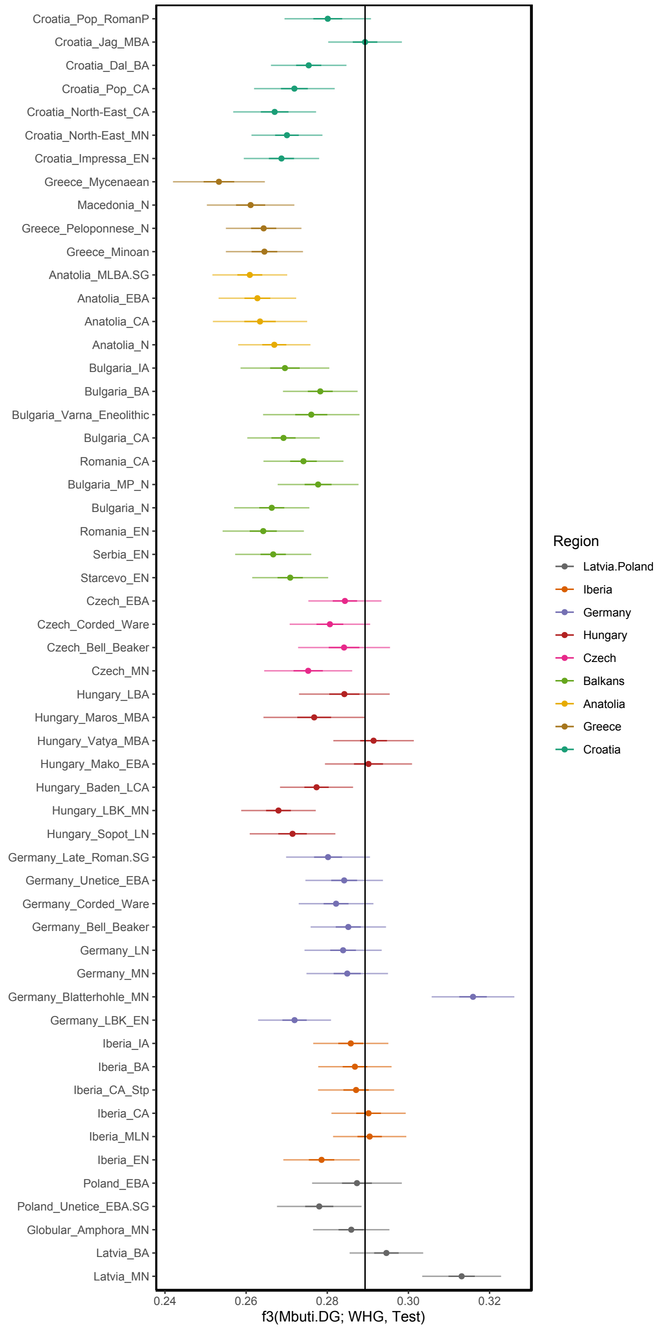

Supplementary Figure S7. Shared genetic drift between ancient populations and WHG. Plot showing shared genetic drift of ancient groups with WHG, using a minimum of 10,000 overlapping SNPs, as measured by the *f*-statistic f_3(Mbuti.DG; WHG, Test) (data in Supplementary Table S3). Groups/individuals are ordered broadly chronologically within colour-coded geographic regions. One and three standard error bars are shown. The vertical solid line indicates the f_3 value for shared drift between WHG and Croatia_Jag_MBA. Points to the left of the line indicate groups harbouring greater shared drift with WHG than Jagodnjak, and points to the right of the line indicate groups that share less genetic drift with WHG than Jagodnjak.

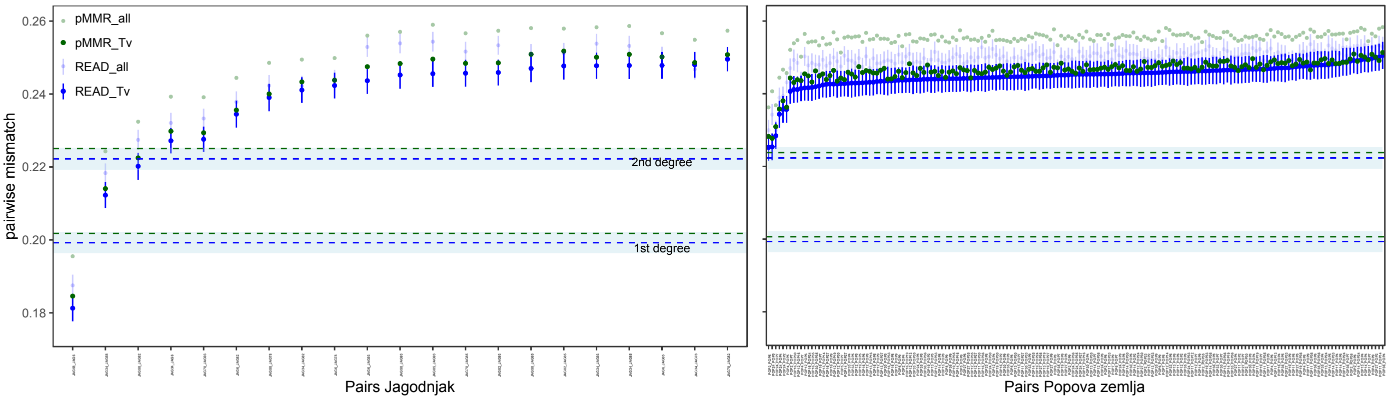

Supplementary Figure S8. Pairwise mismatch rates. Plot of pairwise mismatch rates between pairs of newly-reported individuals to determine relatedness using READ (blue points) and pMMRCalculator (green points) (data in Supplementary Table S9). The left panel shows results for Croatia_Jag_MBA and the right panel for Croatia_Pop_MN. Filled points indicate results for the genotype dataset filtered on transversion-only SNPs (READ_Tv and pMMRC_Tv in the legend) (see Methods). Standard error bars indicating one standard error, calculated by READ, are shown for READ results. Horizontal dashed blue lines indicate upper limit of first and second degree relatedness as determined by READ, with shading indicating the standard error. Green dashed lines indicate these upper limits based on results calculated by pMMRCalculator. Additionally, translucent points show results for the dataset containing both transversion and transition SNPs (READ_all and pMMRC_all in the legend). Points lower down along the y-axis indicate a lower mismatch rate and therefore a closer consanguineous relationship. See Methods for further discussion about these methods.

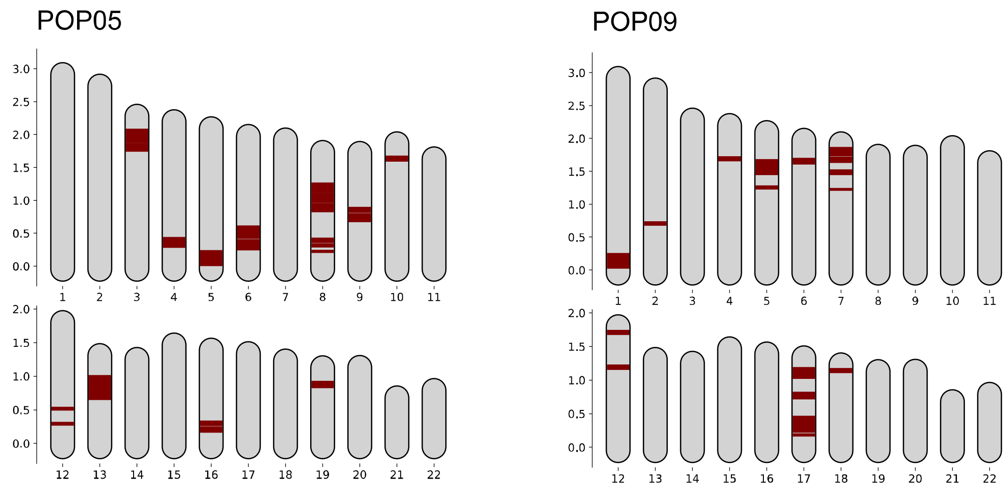

Supplementary Figure S9. Inferred Runs of Homozygosity. Karyograms showing the chromosomal distribution of runs of homozygosity for the two individuals with the highest inferred ROH (data in Supplementary Table S10).

Other supplementary materials include Supplementary Data file S1 (containing Supplementary Tables S1-S11 as tabs in Microsoft Excel spreadsheet)

Legends for Supplementary Data file S1:

Supplementary Table S1. Sample information. Summary of samples analysed in this study.

Supplementary Table S2. Ancient samples. List of main published ancient genomes and groupings included in analyses.

Supplementary Table S3. Outgroup f3-statistics. Sets of outgroup f_3-statistics testing shared genetic drift between new Croatian groups and ancient and present-day populations.

Supplementary Table S4. qpAdm and qpWave. Results for qpWave and qpAdm analyses are based on the set of outgroups Mbuti.DG, Ust_Ishim_HG_published.DG, Ethiopia_4500BP.SG, Russia_MA1_HG.SG, Italy_Villabruna, Papuan.DG, Onge.DG and Han.DG.

Supplementary Table S5. Admixture dating. Results for estimated date of admixture using DATES between Anatolia_N and WHG for Croatia_North-East_MN and Croatia_Jag_MBA.

Supplementary Table S6. Cladality test. Sets of f4-statistics testing the amount of allele sharing between I3499 and POP39 and other ancient groups.

Supplementary Table S7. f4-statistics. Sets of f4-statistics testing the amount of allele sharing between the new ancient Croatian individuals and other ancient and present-day West Eurasian groups.

Supplementary Table S8. Sex-biased admixture. qpAdm admixture weights calculated for the autosome and the X chromosome and resulting Z scores to test for sex-biased ancestry.

Supplementary Table S9. Biological kinship. Pairwise mismatch rates calculated with READ (with standard errors, READ_SE) and pMMRCalculator for a dataset containing all SNPs overlapping with the 1240K panel (all SNPs) and another filtered for transversion-only SNPs.

Supplementary Table S10. ROH. Results of inbreeding analysis showing for each individual the maximum length of a run of homozygosity (roh) in centiMorgans (cM) (max_roh), and the number (n_roh) and sum length (sum_roh) of runs of homozygosity above 4,8,12 and 20cM.

Supplementary Table S11. Functional SNPs. SNP analysis for selected phenotypic traits. For each individual, the number of reads supporting the reference and alternative allele on the + strand of the hg19 build of the human reference genome are shown at each SNP position, with the effect allele indicated in bold.
